# Deep sampling of gRNA in the human genome and deep-learning-informed prediction of gRNA activities

**DOI:** 10.1038/s41421-023-00549-9

**Published:** 2023-05-16

**Authors:** Heng Zhang, Jianfeng Yan, Zhike Lu, Yangfan Zhou, Qingfeng Zhang, Tingting Cui, Yini Li, Hui Chen, Lijia Ma

**Affiliations:** 1 Center for Genome Editing, Westlake Laboratory of Life Sciences and Biomedicine, Hangzhou, Zhejiang China; 2 School of Life Sciences, Westlake University, Hangzhou, Zhejiang China; 3 Institute of Biology, Westlake Institute for Advanced Study, Hangzhou, Zhejiang China; 4 AIdit Therapeutics, Hangzhou, Zhejiang China

**Keywords:** Bioinformatics, Non-homologous-end joining

## Abstract

Life science studies involving clustered regularly interspaced short palindromic repeat (CRISPR) editing generally apply the best-performing guide RNA (gRNA) for a gene of interest. Computational models are combined with massive experimental quantification on synthetic gRNA-target libraries to accurately predict gRNA activity and mutational patterns. However, the measurements are inconsistent between studies due to differences in the designs of the gRNA-target pair constructs, and there has not yet been an integrated investigation that concurrently focuses on multiple facets of gRNA capacity. In this study, we analyzed the DNA double-strand break (DSB)-induced repair outcomes and measured SpCas9/gRNA activities at both matched and mismatched locations using 926,476 gRNAs covering 19,111 protein-coding genes and 20,268 non-coding genes. We developed machine learning models to forecast the on-target cleavage efficiency (AIdit_ON), off-target cleavage specificity (AIdit_OFF), and mutational profiles (AIdit_DSB) of SpCas9/gRNA from a uniformly collected and processed dataset by deep sampling and massively quantifying gRNA capabilities in K562 cells. Each of these models exhibited superlative performance in predicting SpCas9/gRNA activities on independent datasets when benchmarked with previous models. A previous unknown parameter was also empirically determined regarding the “sweet spot” in the size of datasets used to establish an effective model to predict gRNA capabilities at a manageable experimental scale. In addition, we observed cell type-specific mutational profiles and were able to link nucleotidylexotransferase as the key factor driving these outcomes. These massive datasets and deep learning algorithms have been implemented into the user-friendly web service http://crispr-aidit.com to evaluate and rank gRNAs for life science studies.

## Introduction

Clustered regularly interspaced short palindromic repeat (CRISPR) technology is revolutionizing biological and clinical studies because of its efficiency, accuracy, and flexibility of gene editing, which allows genes to be precisely edited or regulated in a wide range of applications. Because of its revolutionary nature, many attempts have been undertaken to improve performance at the levels of developing new enzymes and optimizing the design and selection of guide RNAs (gRNAs).

The selection of gRNAs depends on a thorough understanding of the connections between the gRNA sequence and gRNA performance, typically reflected by the cleavage activities at the desired and undesired sequences and the ultimate mutational profiles. In recent times, computational models have been developed to predict gRNA capabilities from massive experimental datasets, such as CRISPR screening and synthetic gRNA-target sequence libraries. Models established from the CRISPR screening involve observations of the phenotypic changes in gene-edited cells, which cannot fully and directly represent the capabilities of gRNAs^1–5^. By contrast, the high-throughput library of synthetic gRNA-target pairs allows direct investigations of in vivo gRNA target sequences in cell lines, although primary cells are more therapeutically relevant.

In previous studies, ~10,000 to ~50,000 synthetic gRNA-target pairs were applied to quantify the on-target efficiency, off-target specificity, or repair outcomes of gRNAs for various CRISPR enzymes. However, it is unclear whether datasets of this scale are at the sweet spot to establish a deep learning model to predict gRNA capability. For instance, the human genome has 6 × 10^8^ potential gRNAs with NGG PAM, which results in 0.002%–0.009% coverage for gRNAs sampled in earlier studies. Given this range of sampling, the computational predictions and experimental measurements of gRNA cleavage activities had a Spearman correlation of ~0.8 in the cell types used to create the training datasets. More datasets from different cell types will likely need to be gathered, though, as the generalization performance of prediction models are currently being studied. Therefore, it will be beneficial to pinpoint the sweet spot of the model performance given manageable size of experiments. In addition, a deep sampling of gRNAs from at least one kind of cell will help us better grasp the advantages of the synthetic gRNA-target library strategy, having said that, we determined if and how much more the prediction score could be improved.

Computational models built on a range of datasets have predicted individual characteristics of the multi-dimensional gRNA capacity. However, the measurements on various assessment cassettes are inconsistent between studies due to differences in the designs of the gRNA-target pair constructs, and there has not yet been an integrated investigation that concurrently focuses on multiple facets of gRNA capacity. In this study, we analyzed the DNA double-strand break (DSB)-induced repair outcomes and measured SpCas9/gRNA activities at both matched and mismatched locations using 926,476 gRNAs covering 19,111 protein-coding genes and 20,268 non-coding genes. We developed machine learning models to forecast the on-target cleavage efficiency (AIdit_ON), off-target cleavage specificity (AIdit_OFF), and mutational profiles (AIdit_DSB) of SpCas9/gRNA from a uniformly collected and processed dataset by deep sampling and massively quantifying gRNA capabilities in K562 cells. In addition, we looked into the best data size to create high-performance models for later use. We also discovered a crucial marker, nucleotidylexotransferase (DNTT) expression, which we used to direct model choice and forecast SpCas9/gRNA mutational patterns specific to different cell types. As a result, our study improved our understanding of how well the synthetic gRNA-target library could improve the thorough knowledge of SpCas9/gRNA activities. Furthermore, the three high-performing models and the web tool http://crispr-aidit.com should considerably enhance the capacity to predict specific gRNA-related editing outcomes across various cell types.

## Results

### A library comprising 740k synthetic gRNA-target pairs reveals SpCas9/gRNA cleavage activity in K562 cells

**Fig. 1:**
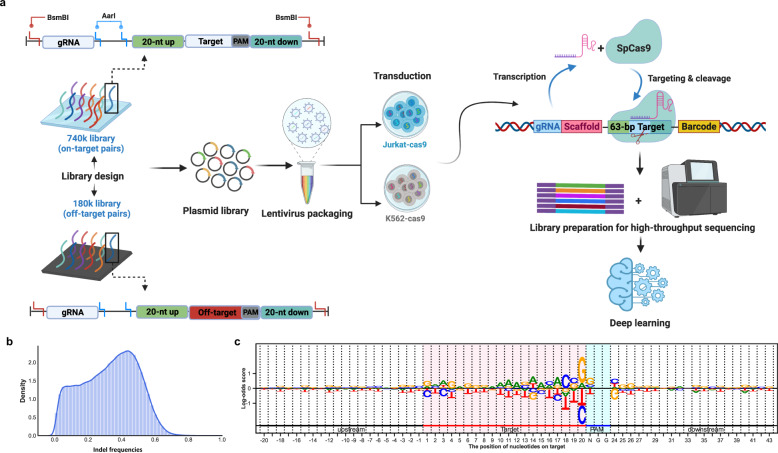
High-throughput quantification of SpCas9/gRNA cleavage activity in vivo. **a** Flowchart of the experimental design used to generate data from the 740k gRNA-target library and 180k gRNA-off-target library. The 740k library comprised 743,344 gRNA sequences, and their corresponding target sequences were selected and combined with the Brunello, GecKOv2 AB, Sabatini, TorontoKoV3, and YusaKoV1 libraries derived from 19,111 protein-coding genes and 20,268 non-coding genes. Each target sequence comprised a 23-nt (including a 3-nt PAM sequence) target sequence, its 20-nt upstream region and its 20-nt downstream region (according to the genomic context). The 180k library comprised 183,132 gRNA-off-target pairs. **b** The distribution of the SpCas9/gRNA activities in K562 cells. *x*-axis: indel frequencies. **c** The nucleotide preferences of SpCas9/gRNA cleavage activity in the target sequences and context sequences in the flanking genomic regions. At each position along the *x*-axis, the gRNAs are ranked in descending order according to their indel frequencies. The percentage of a particular nucleotide in the first quartile at each position was divided by its percentage in the fourth quartile. The log2-transformed relative ratio is denoted as the log-odds score (*y*-axis), representing relative abundance in the 1st and 4th quartiles.

Extending previous studies based on tens of thousands of gRNA sequences, we designed a 740k gRNA-target pair library corresponding to 26 gRNAs per protein-coding gene and 12 gRNAs per non-coding gene (Fig. 1a). The library was a combination of the gRNAs that we newly designed for this study and other published libraries, including Brunello^6^, GecKOv2^7^, Sabatini^8^, TorontoKoV3^9,10^, and YusaKoV1^11^ (Supplementary Table S1). This library represents ~0.16% of all gRNAs with NGG PAMs in the human genome (higher than the 0.002%–0.009% coverage in previous studies). The assessment cassette for each gRNA included the gRNA itself and its 63-nucleotides (nt) putative genome target, which was composed of a 20-nt upstream region, 20-nt target sequence, 3-nt NGG PAM, and 20-nt downstream region. The assessment cassettes were cloned into a lentivirus backbone with a 20-nt random nucleotide barcode (BC) sequence, which was incorporated to eliminate confounding issues stemming from processes such as plasmid propagation and polymerase chain reaction (PCR) amplification (see Materials and methods).

Under the assumption that relative indel frequencies and indel frequency ranks are preserved when different amounts of lentivirus are transduced^12^, transduction was conducted at a multiplicity of infection (MOI) of 5 to achieve high cell-to-library coverage while generating a cell population that was manageable in size. The cells were harvested at day 3.5 post-transduction, and the indel frequencies of all gRNAs were assessed by high-throughput sequencing (HTS). We filtered those gRNAs with read numbers ≥ 200 and BC numbers ≥ 10 (see Materials and methods) and plotted their indel frequencies against their paired target sequences (Fig. 1b; Supplementary Fig. S1a). The frequency spectra collectively showed that the indel rates were widely distributed (median: 0.34) and were strongly positively correlated between biological replicates (Pearson r = 0.93) (Supplementary Fig. S1b), supporting the notion that the results of our indel frequency quantification were robust and reproducible.

To gain an overview of the nucleotide preferences of cleavage activity alongside the gRNA targets and their flanking regions, we aggregated all target sequences in the 740k library and generated a nucleotide preference map of SpCas9/gRNA cleavage activity in K562 cells. We used the odds ratio of the nucleotide frequency between the favored and disfavored nucleotides at each position to represent nucleotide preferences across the 63-base pairs (bp) target region (Fig. 1c). This analysis showed that the four nucleotides adjacent to the PAM sequence exerted obviously stronger impacts on gRNA cleavage activities than other nucleotides and a guanine positioned immediately adjacent to the 5′ end of the PAM was more likely to increase cleavage than other nucleotides at these positions^13–16^. These results echoed the nucleotide preferences concluded from previous smaller-scale gRNA-target paired libraries and further verified the fidelity of cleavage activity with more than 20-fold expansion in data size.

### Building a machine learning model to predict SpCas9/gRNA cleavage activity

To develop prediction models for the on-target activity of gRNAs, we initially compared nine machine learning algorithms, comprising seven conventional algorithms (Lasso regression, ridge regression, elastic regression, random forest, gradient boosting decision tree (GBDT), extreme gradient boosting (XGBoost) and multilayer perceptron (MLP) algorithms) and two deep learning-based algorithms (convolutional neural network (CNN) and recurrent neural network (RNN) algorithms). We split the cleavage activity data contained in the 740k library into a training dataset (90%) and a test dataset (10%). We randomly picked 10,000 gRNA sequences and used their indel frequencies to optimize the model hyperparameters using Hyperopt (see Materials and methods).

**Fig. 2:**
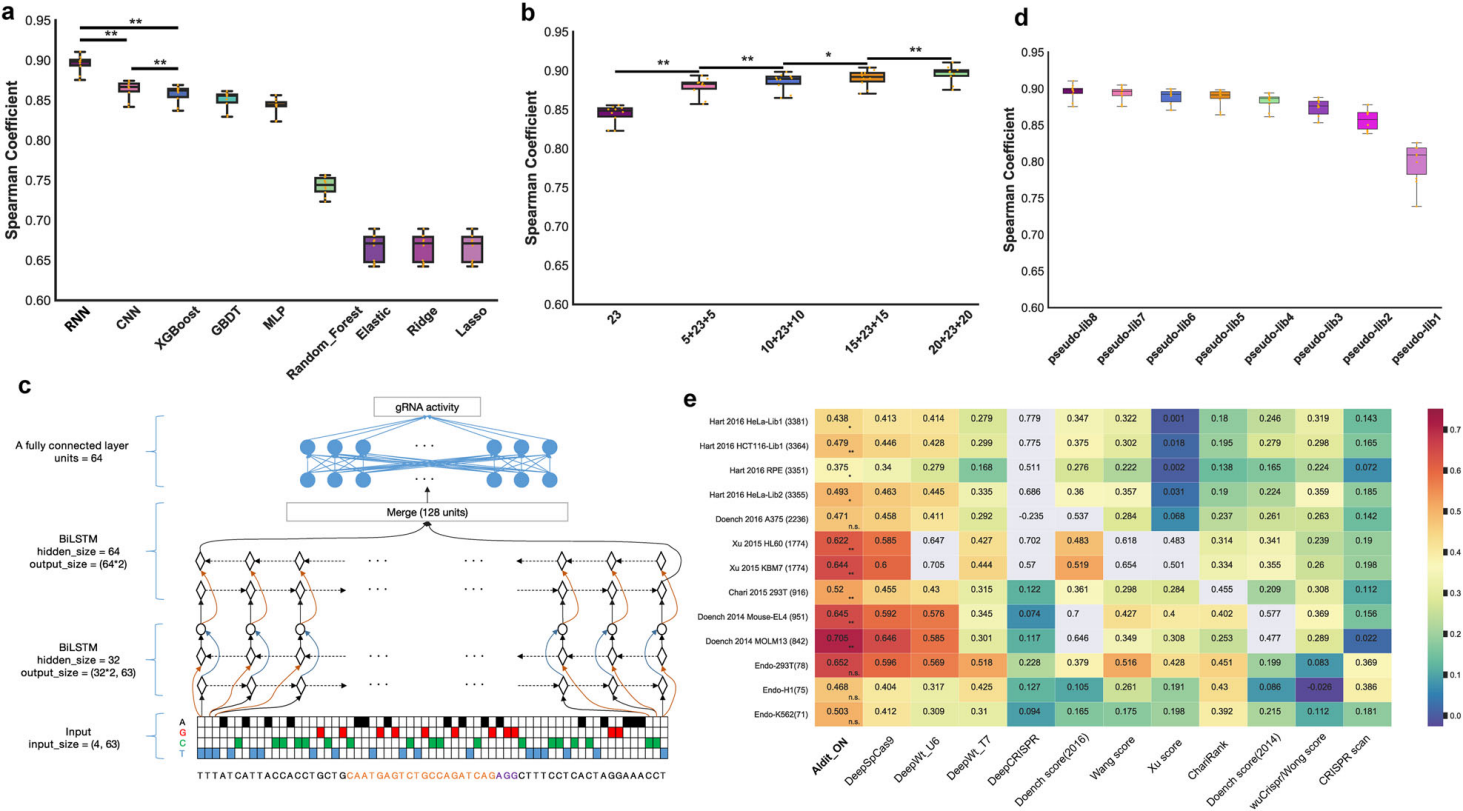
Building AIdit_ON model to predict Cas9/gRNA cleavage activity. **a** Comparison of the prediction performances achieved by different algorithms for the cleavage activities of SpCas9/gRNA. The models were trained by 10-fold cross-validation. Each dot represents the Spearman correlation between the measured indel frequencies and the predicted efficiencies from 10-fold cross-validation. *P < 0.05 and **P < 1e–5 indicate statistically significant differences between two deep learning-based approaches (between the deep learning-based approaches and the best conventional machine learning-based algorithm) according to Steiger’s test. **b** The influence of the input sequence length on model performance. The 10-fold cross-validation process for the RNN models was performed on training datasets with 23-bp target sequences only (23), and with additional downstream and upstream sequences of varying lengths (with the bp lengths presented before and after the target sequence length, respectively). Each dot represents the Spearman correlation coefficient between the measured indel frequencies and the predicted efficiencies from the 10-fold cross-validation (*P < 0.05 and **P < 1e–5, Steiger’s test). **c** Schematic representation of the workflow of the AIdit_ON model for predicting gRNA activity. The input of AIdit_ON was one-hot encoded 63 bp sequences. Number of hidden size of the first BiLSTM layer and the second BiLSTM layer was 32 units and 64 units, respectively. And the merge mode of both these two BiLSTM layers was elementwise concatenation. The second BiLSTM layer outputted the merge of last hidden state of its forward and backward layers which was 128 units and was inputted a fully connected layer with 64 units. Finally, the fully connected layer outputted gRNA activity. **d** The influence of the input data scale on model performance. The 10-fold cross-validation process for the RNN models was performed on training datasets in eight pseudolibraries with gradually increasing library sizes from pseudolib1 to pseudolib8. **e** Heatmap of the Spearman correlation coefficients between the measured indel frequencies and the predicted efficiencies across twelve models (columns) for various datasets (rows). Abbreviated information about the datasets, including their years of publication, cell types, and numbers of included gRNAs, is provided in the row labels. The column labels represent the names of the computational models included in this benchmark. The Spearman correlation coefficients between the reported or measured indel frequencies and the predicted efficiencies are color-coded. The gray blocks in the heatmap indicate that the model was evaluated against its own training datasets. The previously reported models included DeepSpCas9^13^, DeepWt_U6^14^, DeepWt_T7^14^, DeepCRISPR^17^, the Doench score^6^, the Wang score^2^, the Xu score^16^, ChariRank^5^, the Doench score^18^, the wuCrispr/Wong score^20^, and CRISPR scan^1^. The statistical significance determined by Steiger’s test is shown between the two best models for each dataset (*P < 0.05, **P < 1e–10; n.s. represents not significant).

When we trained the seven conventional models and two deep learning models on the training dataset using 10-fold cross-validation, we found that the two deep learning frameworks (RNN and CNN) performed better in predicting on-target activity than the conventional algorithms (Fig. 2a). The RNN was the best-performing of all the tested algorithms, with Spearman correlation coefficients between the measured indel frequencies and the predicted scores ranging from 0.875 to 0.911 (median: 0.898) (Fig. 2a). When the trained models were applied based on the test dataset, the RNN again performed best among all the models (Supplementary Fig. S2). Thus, we selected the RNN model built from the 740k library data collected in K562 cells for further analyses.

An analysis in which the lengths of the flanking sequences were gradually extended from the central target showed that the performance of the RNN model continued to increase with the length of input sequences (Fig. 2b). The greatest effect was obtained with a flanking sequence of ±5 bp, which significantly increased the Spearman coefficient from 0.848 (without any flanking sequence) to 0.883. And inclusion of flanking sequence of ±10 bp, ±15 bp, and ±20 bp supported length-related contributions of the sequence context (Fig. 2b). We further dissected the contributions derived from the upstream and downstream flanking sequences and found that the downstream gRNA sequence contributed to a greater extent than the upstream sequence (Supplementary Fig. S3). Hence, we included 63-bp sequences (23 bp plus 20 bp from both upstream and downstream) as input sequences for the RNN model and named it “AIdit_ON”, indicating its function of predicting the on-target SpCas9/gRNA cleavage activities (Fig. 2c).

The strong detection performance of AIdit_ON trained on the 740k library encouraged us to investigate the performance improvements achieved with increases in the data scale. The requirement for cell-to-library coverage makes it challenging to apply large-scale synthetic library to primary cells, which are more relevant to medical applications such as CRISPR therapy. Expanding the pool of cell types with available high-quality datasets might allow us to better answer that question. Achieving this goal may be limited, however, by the lack of guidelines regarding the “sweet spot” in the size of the datasets used for establishing an effective model at a manageable experimental scale. To this end, we split the 740k library into eight subpools (see Materials and methods). Starting with one subpool as the first pseudolibrary, we generated the second pseudolibrary by adding one more subpool. By sequentially adding more subpools, we generated eight pseudolibraries (pseudolib1–8) representing a gradually increasing data scale. And the pseudolib8 is the same to what we used to build the AIdit_ON model. We trained the RNN model for each of the pseudolibraries using 10-fold cross-validation and calculated the Spearman correlation coefficients between the measured indel frequencies and the predicted scores. As pseudolibrary size increased, the prediction performance of the eight models gradually increased from 0.810 to 0.898 (Fig. 2d). We noted that the performance improvement benefited the most from pseudolib1 (median: 0.810, ~40k gRNAs) to pseudolib4 (median: 0.887, ~173k gRNAs). Although the performance improvement trend continued to increase when the size of pseudolibraries increased, the curve became relatively flat starting with the inclusion of pseudolib5 (median: 0.891, ~220k gRNAs). This pseudolibrary analysis indicated that the increase in the data scale of the synthetic gRNA-target library contributed to model performance. However, the performance gain became flattened after more than 220k gRNAs were included.

### Favorable Aldit_ON performance at endogenous sites and in various further cell types

To investigate whether the AIdit_ON model established on K562 cells is applicable to other cell types, we applied the 740k library to Jurkat cells with stable Cas9 expression following the same experimental protocol. As expected, the Jurkat dataset also exhibited a uniform indel frequency distribution and high reproducibility between biological replicates (Supplementary Figs. S1b, S4). The Spearman correlations between the replicates of K562 and Jurkat cells ranged from 0.73 to 0.82, suggesting similar SpCas9/gRNA cleavage activities in these two cell lines. However, the correlations between cell types are still lower than that between biological replates of one cell type, indicating some gRNAs behaved differently between the K562 and Jurkat cells. Further analysis on these gRNAs demonstrated distinct nucleotide preferences around the SpCas9/gRNA cleavage site (Supplementary Fig. S5), suggesting unknown cell-type specific factor, together with distinct nucleotides around the cleavage site, contributes to the cleavage activities of SpCas9. Interestingly, when the AIdit_ON model was applied to predict the cleavage activities of gRNAs in Jurkat cells, the Spearman correlation was 0.77 (Supplementary Fig. S6). This prediction score was within the range of Spearman correlations obtained between the two cell types, indicating that AIdit_ON shows good performance generalization for synthetic datasets.

To further examine the generalization performance of the models among more cell types, we applied the AIdit_ON model and eleven published models for prediction based on publicly available datasets that have been included in multiple benchmarks on prediction models of cleavage activities. These datasets represented SpCas9/gRNA cleavage activities inferred from CRISPR screenings that conducted in cell types including HeLa, HCT116, RPE-1, A375, HL60, KBM-70, HEK293T, Mouse-EL4, and MOLM13^1,2,5,6,13^^,14^^,16–21^. To eliminate systematic variations and prevent overfitting, we used ten datasets with more than 100 gRNAs (excluding gRNAs overlapping with those in our training set) driven by the U6 promoter from published datasets. Although the obtained prediction scores fell within a larger range (0.375–0.705), the AIdit_ON model outperformed all other models across all public datasets (Fig. 2e).

Next, to examine the prediction performance of the AIdit_ON model at endogenous sites, we conducted CRISPR knockout with 96 randomly selected gRNAs in K562, HEK293T, and human embryonic stem cells (H1) to investigate the generalizability of the models. We quantified the corresponding indel frequencies in the three cell lines (Endo-293T *n* = 78; Endo-K562 *n* = 75; Endo-H1 *n* = 71) (Supplementary Fig. S7 and Table S2). The Spearman correlation coefficients between the model predictions and the experimental quantification results were 0.65 for HEK293T cells, 0.5 for K562 cells, and 0.47 for H1 cells (Supplementary Fig. S8). Notably, the AIdit_ON model again outperformed the other models in all three endogenous datasets, which emphasized that its good performance was generalized (Fig. 2e).

### The AIdit_ON model predicts SpCas9/gRNA cleavage activities in human primary cells for CRISPR therapy

To facilitate gRNA selection for biomedical researchers, we established a public web service (crispr-aidit.com) embedded with the AIdit_ON model. With this service, people can score and rank gRNAs for their genes of interest (GOIs) by experimentally quantified or predicted efficiency. A list of gRNAs with predicted efficiency could be obtained by entering a gene name, a stretch of sequence, or a FASTA file with sequences. As an example, we searched for and ranked gRNAs corresponding to the pyruvate kinase, liver, and red blood cell (*PKLR*) gene locus. Segovia and colleagues recently reported an ex vivo CRISPR therapy in a clinically applicable system using SpCas9/gRNA to treat pyruvate kinase deficiency (PKD)^22^. The gRNA that they ultimately chose over the other candidates matched the top1 candidate predicted by AIdit_ON (Supplementary Fig. S9a).

The K562 cells were often used to screen for gRNAs with high editing efficiency before ex vivo CRISPR gene editing was conducted in human hematopoietic stem cell (HSC). In some reported studies, we found that AIdit_ON successfully predicted the top1 candidates for different genomic loci, including *α-Globin*^23^, *FOXP3*^24^, and *HBB*^25^, and the correlation of the model predicted and experimental measured indel efficiencies are high (Supplementary Fig. S9b–d).

Besides HSC, ex vivo CRISPR therapy also expands the way to engineer T cells for CAR-T cell therapy. And the AIdit_ON model performed well in predicting the editing efficiencies of gRNAs targeting *B2M*, *TRAC*, and genomic loci in human primary T cells^26^ (Supplementary Fig. S9e).

### The 740k library reveals insertion-preferred repair outcomes in Jurkat cells but not K562 cells

SpCas9 generates non-random and reproducible mutational profiles depending on the given sequence context. The datasets of 740k library in both K562 and Jurkat cells allowed us to further examine DSB-induced repair outcomes in these two cell types. Different from the cleavage activity that could be directly used to represent the gRNA property, a metrics is firstly needed to quantify the repair outcome of each gRNA. We compared the repaired sequences to the wild-type (WT) sequences and sorted the repair outcomes from three dimensions, including the repair type (insertion or deletion), the repair location, and the lengths of the repaired sequences. We comprehensively defined 631 repair categories according to the above three dimensions and calculated the percentages of categories for each gRNA, which was used as the metrics to represent its repair outcomes. The median Pearson coefficients of the biological replicates were 0.954 for K562 cells and 0.920 for Jurkat cells, suggesting that the defined repair categories faithfully represented the high reproducibility between biological replicates as we have demonstrated in the analysis of cleavage activities (Supplementary Fig. S10). However, the median Pearson coefficient between K562 and Jurkat cells is only 0.544. The symmetric Kullback Leibler (KL) divergence, which is a metrics for the relative entropy of probability distribution and has been used to quantify similarity of repair outcomes, also supported the high reproducibility between replicates and relative low similarity between the K562 and Jurkat cells (Supplementary Fig. S10).

**Fig. 3:**
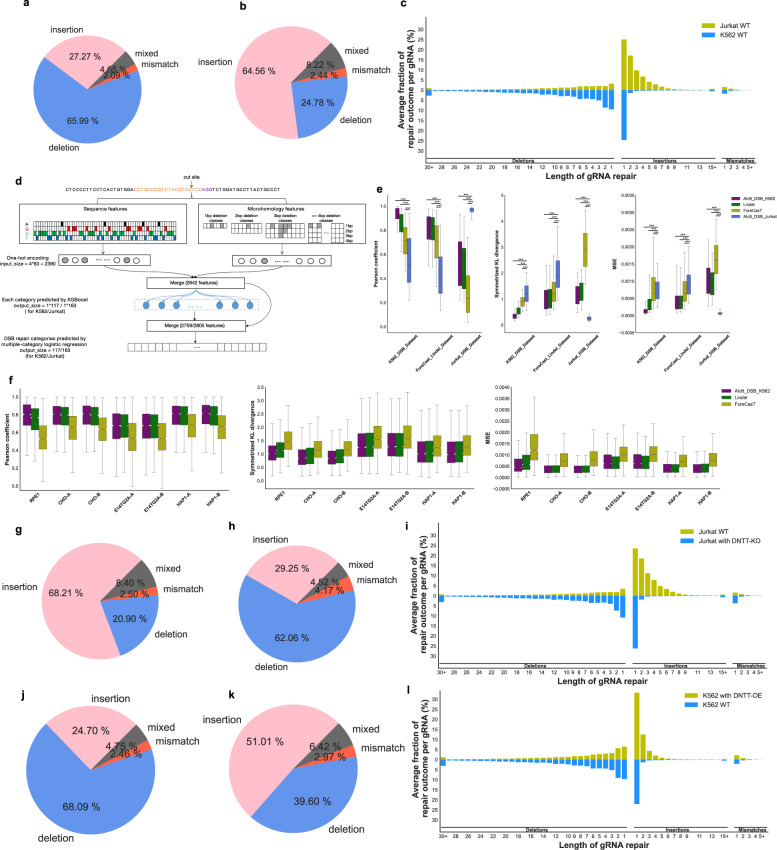
DSB-induced repair outcomes are diverse and biased. **a**–**c** Distribution of SpCas9-induced DSB repair outcomes in K562 and Jurkat cells. The pie charts show the average occurrence percentages of insertions, deletions, mismatches, and mixed mutations per gRNA in K562 (**a**) and Jurkat (**b**) cells. The bidirectional bar plots show the average fraction of each repair category per gRNA in K562 and Jurkat cells (**c**). **d** Schematic representation of the workflow of the AIdit_DSB models for repair outcome prediction for SpCas9/gRNA. The sequence and microhomology features were extracted from the 63-bp input sequences and merged into 2642 features. The input of AIdit_DSB was a merge datasets of these 2642 features and XGBoosts predictions, which came from 117/163 XGBoost models fitting each category of DSB-induced repair outcomes for K562/Jurkat, respectively. AIdit_DSB finally predicted 117 and 163 DSB repair categories through multiple-category logistic regressions for K562 and Jurkat, respectively. **e** Comparison of model performance regarding the prediction of DSB-induced repair outcomes. The benchmark was generated based on four different models (AIdit_DSB_K562, Lindel, ForeCast, and AIdit_DSB_Jurkat) across three datasets (K562, ForeCast_Lindel, and Jurkat). Three metrics were compared, including Pearson correlation (left), symmetrized KL divergence (middle), and MSE (right). **f** Comparison of model performance in predicting DSB-induced repair outcomes. The benchmark was generated based on three different models (AIdit_DSB_K562, Lindel, and ForeCast) across seven datasets (*x*-axis). Three metrics were compared, including the Pearson correlation (left), symmetrized KL divergence (middle), and MSE (right). **g**–**i** Distribution of SpCas9-induced DSB repair outcomes in Jurkat WT cells and Jurkat cells with *DNTT* knockout (*DNTT*-KO). Both the Jurkat WT cells and the *DNTT*-KO Jurkat cells were transduced with the library-3, one of the eight subsets of the 740k library. The pie charts show the average occurrence percentages of insertions, deletions, mismatches, and mixed mutations per gRNA in Jurkat WT cells (**g**) and Jurkat cells with *DNTT*-KO (**h**). The bidirectional bar plots show the average fraction of each repair category per gRNA in Jurkat WT cells and Jurkat cells with *DNTT*-KO (**i**). **j**–**l** Distribution of SpCas9-induced DSB repair outcomes in K562 WT cells and K562 cells with *DNTT* overexpression (*DNTT*-OE). Both the K562 WT cells and the *DNTT*-OE K562 cells were transduced with the library-3, one of the eight subsets of the 740k library. The pie charts show the average occurrence percentages of insertions, deletions, mismatches, and mixed mutations per gRNA in K562 WT cells (**j**) and K562 with *DNTT*-OE cells (**k**). The bidirectional bar plots show the average fraction of each repair category per gRNA in K562 WT cells and K562 cells with *DNTT*-OE (**l**).

A further analysis showed that 65.99% of the SpCas9/gRNA-induced DSBs were repaired as deletions in K562 cells (Fig. 3a). By contrast, insertions dominated in Jurkat cells (64.56% of DSBs; Fig. 3b, c). Previous studies in several cell types (e.g., HEK293T, K562, mESC, Human iPSC, et al.) have reported that deletions account for the most dominant proportion of DSB repairs^14,27,28^. Hence, we wondered why Jurkat cells showed a preference for insertions instead of deletions, and whether we could use that information to guide model selection for DSB repair outcome prediction. We therefore formulated the prediction of repair outcomes as a multiclass regression problem and aggregated the 631 repair categories to increase the robustness of the models by merging nearby categories according to the principle of category proximity (Supplementary Fig. S11 and Tables S3, S4; see Materials and methods). We derived the 63-bp target sequence and microhomology features as inputs and adopted an ensemble learning strategy to train the models. We first trained an individual XGBoost model for each repair category to predict the category frequencies, which were plugged into the final training models as additional features. The final models were trained using a multiclass logistic regression algorithm, in which the categorical cross-entropy was used as a loss function (Fig. 3d). The model trained on the K562 data was named AIdit_DSB_K562, and the model trained on the Jurkat data was named AIdit_DSB_Jurkat.

We compared the generalization performance of our models with that of ForeCasT^27^ and Lindel^28^ based on three datasets using three evaluation metrics: the Pearson correlation coefficient, mean square error (MSE), and symmetric KL divergence (Supplementary Fig. S12). Across all datasets and metrics, the AIdit_DSB_K562 model outperformed the Lindel and ForeCasT models (Fig. 3e; Supplementary Fig. S12). The superior performance of AIdit_DSB_K562 was also demonstrated based on other public datasets collected from cell lines, including REP1, CHO, E14TG2A, and HAP1cells^27^ (Fig. 3f). However, AIdit_DSB_Jurkat showed very low prediction efficacy for non-Jurkat data, whereas the other models showed poor prediction scores for the Jurkat data.

### DNTT expression drives insertion-preferred repair outcomes

We hypothesized that a unique property of Jurkat cells might be responsible for the insertion-dominant repair outcomes we observed in these cells, and the identification of that property might help with the selection of repair outcome prediction models. To test this hypothesis, we looked up the expression of genes that were involved in DNA repair pathways from the Human Protein Atlas database and filtered for those were specifically and highly expressed in Jurkat cells. Hence, we identified *DNTT* as a gene that was more highly expressed in Jurkat cells than in K562 cells (Supplementary Fig. S13,https://www.proteinatlas.org/ENSG00000107447-DNTT/cell+line)^29^.

*DNTT* is a member of the DNA polymerase type-X family and encodes a template-independent DNA polymerase that catalyzes the addition of deoxynucleotides to the 3′-hydroxyl terminus of an oligonucleotide primer^30^. If DNTT is responsible for the insertion preference in Jurkat cells, we reasoned that knocking out *DNTT* in Jurkat cells would change their repair outcomes, making them more similar to those of K562 cells. Therefore, we used a subset of gRNA target cassettes from the 740k library and transduced them into a *DNTT*-KO Jurkat-Cas9 cell line (see Materials and methods). Strikingly, the resulting repair outcome shifted from insertion-dominant to deletion-dominant, which accounted for 60.58% of DSB repairs, similar to what was observed in K562 cells (Fig. 3g–i). Conversely, when we overexpressed *DNTT* in K562-Cas9 cells (*DNTT*-OE K562-Cas9), insertions became the most dominant DSB repair profile (Fig. 3j–l). Collectively, these data demonstrate that the DSB-induced repair outcomes mediated by SpCas9/gRNA were DNTT-dependent.

### Building a machine learning model to predict SpCas9/gRNA off-target activity with a library comprising 180k synthetic gRNA-off-target pairs

**Fig. 4:**
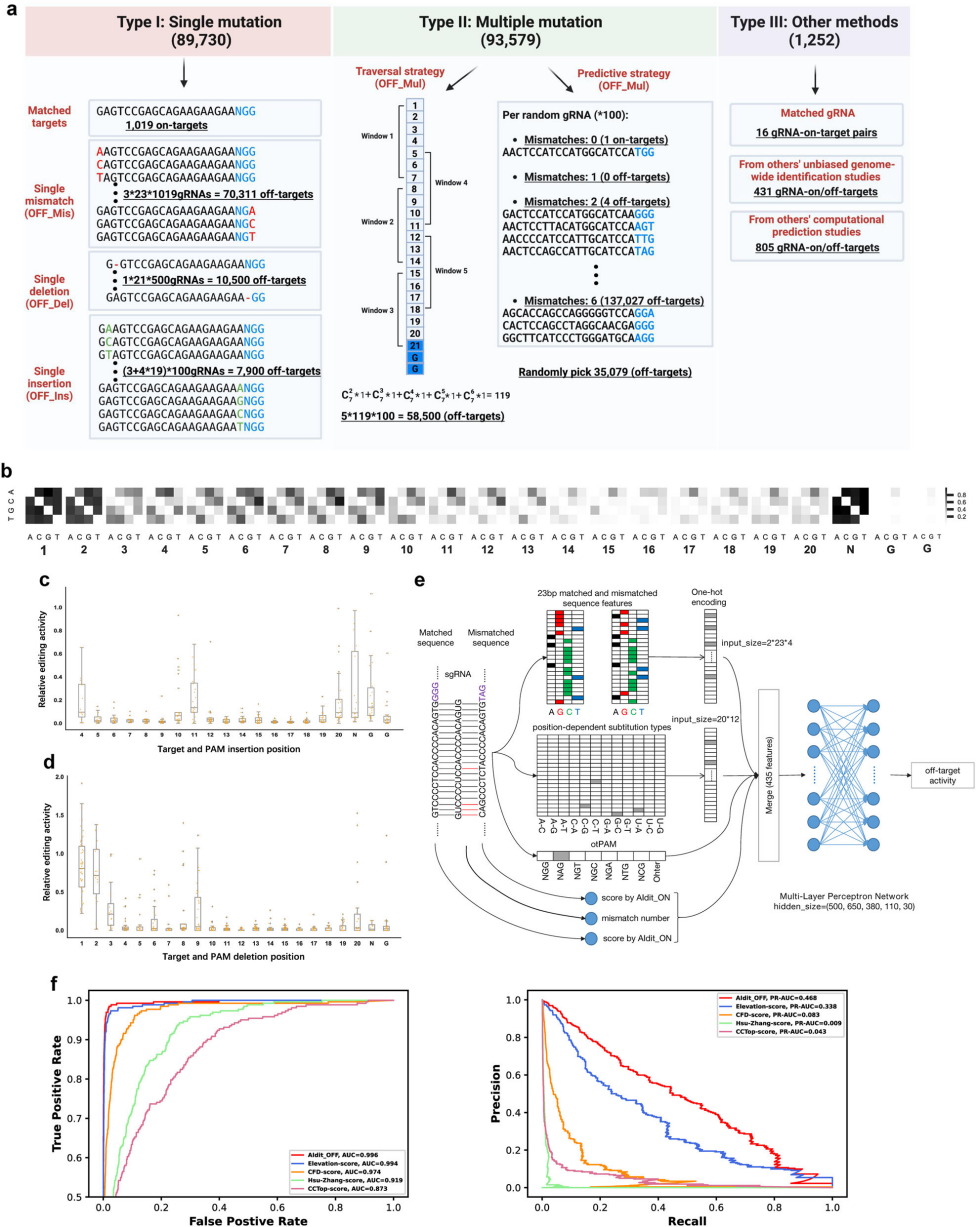
Building AIdit_OFF model to predict SpCas9/gRNA off-target activity. **a** Schematic representation of the sequence design process for the 180k synthetic gRNA-off-target library. The 180k library included 184,561 carefully designed off-target sequences. These sequences included: 89,730 single-mutation off-targets, which could be further divided into single mismatches (OFF_Mis), single deletions (OFF_Del), and single insertions (OFF_Ins); 93,579 off-targets with multiple mismatches (OFF_Mul), which were generated via a traversal strategy and a predictive strategy; and 1252 pairs collected from computational predictions or experimental assays (e.g., GUIDE-seq). The former two groups were utilized to quantify indel frequencies associated with different off-target types at a large scale. The latter group was used to validate our method. **b** Heatmap of average relative indel frequencies between the matched targets and off-targets with 1-bp mismatches. At each position along the target region, the columns represent the nucleotides of the target sequences, and the rows represent the mismatched nucleotides. The relative indel efficiencies are color-coded. **c** Influence of insertion position on off-target sequences with 1-bp bulges. The relative editing activities, which are relative ratios of indel frequencies between the off-target sequences and the corresponding matched targets, were plotted on the *y*-axis. Positions 1–3 were excluded from this analysis due to data filtering. **d** The influence of the insertion position on off-target sequences with deletions. The relative editing activities, which are relative ratios of indel frequencies between the off-target sequences and the corresponding matched targets, were plotted on the *y*-axis. **e** Schematic representation of the workflow of the AIdit_OFF models for indel frequency prediction in off-targets for SpCas9/gRNA. The input of AIdit_OFF included one-hot encoded 23 bp sequence of both matched and mismatched target sequence of gRNAs (184 features), position-dependent substitution types (240 features), the PAM types of targets (8 features), mismatch number and prediction values of AIdit_ON for both matched and unmatched target sequences. These features were merged to serve as the input of a built multilayer perceptron network with five hidden layers whose hidden sizes were 500, 650, 380, 110, and 30, respectively. Finally, the output of the multilayer perceptron network was used to predict off-target activity. **f** Comparison of the model performances in terms of predicting cleavage activities on off-target sequences. The benchmark was conducted on endogenous off-target datasets, which were generated using GUIDE-seq across different models (AIdit_OFF, Elevation_score, CFD score, CCTop score, and Hsu score). Three metrics were compared, including the area under the curve (AUC) (left) for examining the false-positive rate and the area under the precision-recall curve (PR-AUC; right) for examining the recall rate.

Encouraged by the results obtained with the 740k gRNA-target library and the corresponding prediction models, we applied the same strategy to model the off-target activities of SpCas9/gRNA at near-cognate sequences. We devised a 180k gRNA-off-target pair library containing 184,561 sequences, including 89,730 sequences with a single mutation (mismatch, insertion, or deletion) compared to the perfectly matched gRNA target sequence, 93,579 sequences with multiple mismatches, and 1252 sequences previously reported as off-targets based on computational prediction or unbiased genome-wide identification studies (Fig. 4a; Supplementary Figs. S14, S15 and Table S5; see Materials and methods).

The analysis of the relative indel frequencies between the mismatched targets and the perfectly matched targets showed that the bases immediately upstream of the cleavage sites (the 14th to the 17th nucleotide of the target sequence from 5′ to 3′) were the most stringently restricted sequences^31,32^, where only certain types of mismatches allowed (Fig. 4b; Supplementary Fig. S16). A similar analysis on off-target sequences with insertions demonstrated that the insertion of a G nucleotide in the NGG PAM caused a decrease in cleavage activity (Supplementary Fig. S17), which emphasized the impact of the 1st downstream nucleotide that we discovered based on the 740k library data (Fig. 1c). The analysis of the insertion and deletion types of off-targets showed a few “compatible” sites in both cases, at which > 10% cleavage activity was retained when sequences were mutated; however, the positions of those “compatible” sites were different for insertions and deletions (Fig. 4c, d). Together, the results obtained from this 180k gRNA-off-target library enhanced our knowledge of the cleavage activities of SpCas9/gRNA toward nearby cognate sequences.

Benefitting from our off-target library, we built what we believe to be the first deep-learning model for scoring the likelihood of cleavage at off-target sequences (Fig. 4e; see Materials and methods). Among the five tested machine learning algorithms, MLP and XGBoost performed better than Lasso regression, ridge regression, or elastic regression on both the validation and test datasets (Supplementary Fig. S18). We evaluated model generalization performances by comparing the predicted off-targets of 44 gRNAs^21,33–35^ with their verified off-targets provided by using GUIDE-seq and other methods (e.g., DiGenome-seq) (Fig. 4f; Supplementary Fig. S19 and Table S6). The benchmark demonstrated that the MLP score model outperformed the CCTop, Hsu-Zhang score, CFD, and Elevation-score models. Thus, we retained this model and named it the AIdit_OFF model in accord with its prediction function at near-cognate sequences.

The off-target score reflects the likelihood that a predicted off-target site is being cleaved. To better utilize the output scores of the AIdit_OFF model to evaluate and choose favorable gRNAs, we established a minimal off-target score of 0.0069, which is comparable to the minimal cutoff of 0.023 used by the CFD score models^21^. After setting this cutoff, we investigated the accuracy of prediction based on the GUIDE-seq data we obtained for K562 cells. The results showed that AIdit_OFF improved the accuracy of predicting real off-targets by an average of 2.6 times relative to CFD while maintaining slightly higher recall rates, for all evaluation datasets (Supplementary Fig. S20).

## Discussion

Using high-throughput experimental measurements from 926,476 synthetic gRNA-target and gRNA-off-target pairs, we developed three deep learning models (AIdit_ON, AIdit_OFF, and AIdit_DSB) for predicting the cleavage activities, editing specificities, and repair outcomes of SpCas9/gRNA in this study. All three models showed exceptional abilities to forecast SpCas9/gRNA activities.

The AIdit_ON model, which was trained based on datasets generated in K562 cells, attained a reasonably high Spearman correlation coefficient (0.91) for the validation datasets derived from the same cell line. Importantly, the model also showed strong generalization performance for datasets gathered from a variety of other cell types, including data on endogenous sites of H1, Jurkat and HEK293T cells as well as CRISPR screening data targeting endogenous or lentivirus integrated sites in HeLa, HCT116, PRE, A375, HL60, KBM7, HEK293T, Mouse EL4, and MOLM13 cells. In addition, when utilizing the AIdit_ON model to rank gRNAs targeting the *PKLR* gene, a causal gene of PKD, we predicted the best-performing gRNA among a set of candidates using our web service (crispr-aidit.com) as supported by a recent study.

The encouraging Spearman correlation coefficients obtained between the Aldit model and a few other published models showed that it was possible to accurately estimate the cleavage activities of SpCas9/gRNA in a variety of cell types. The inclusion of chromatin accessibility data, a typical cell type-specific feature, did not improve model performance, according to a recent study by Kim and colleagues^13^. Despite being somewhat indirect, it supported the ability of models to anticipate SpCas9/gRNA on-target activities to be generalized.

In addition, we also found that the high generalization performance achieved with the AIdit_ON model did not apply to the AIdit_DSB model. In this instance, we observed insertion-dominant repair outcomes in Jurkat cells while deletion-dominant repair outcomes in K562 cells. We were able to link DNTT, a DNA nucleotidylexotransferase involved in the NHEJ DNA repair pathway, to these distinctive repair patterns. The DSB repair patterns of both cell types were successfully shifted to the opposite way by overexpressing DNTT in K562 cells and knocking out DNTT in Jurkat cells, which indicated that DNTT might be utilized as a marker for picking a repair outcome prediction model.

Our large-scale synthetic gRNA-target and gRNA-off-target libraries allowed us to perform the high-throughput quantification of cleavage activities and repair outcomes in cultured cells. The 740k and 180k gRNA libraries employed in this study represented approximately 0.16% of the known NGG PAM gRNAs across the human genome. This represents a data scale 20 times greater than those in published studies involving machine learning-based modeling. We demonstrated that the model performance greatly improved as the number of gRNAs gradually climbed to ~200,000 by building eight pseudolibraries in silicon with progressively more gRNAs. The performance growth curve, however, flattened when more data were included. This curve could be used to balance the expected model performance and experiment scale as more datasets in more cell types are likely to be created in this field to further improve our capability to forecast the gRNA activities.

We believe that these valuable datasets and high-performance prediction models could contribute to the research community to improve the prediction performance of CRISPR editors. In addition, with a better understanding of their generalization performance, these datasets and prediction models will enhance our ability to master the application of CRISPR technology to both biomedical research and CRISPR therapies.

## Materials and methods

### Oligonucleotide library design

To assemble the library for predicting on-target Cas9/gRNA cleavage activities (Aldit_ON), unique 20-bp sequences with NGG PAM-containing sites were selected by scanning the human genome (hg38), and their off-target scores were calculated according to the algorithm developed by John G. Doench and colleagues^6^. Potential sgRNAs that aligned with the coding sequence (CDS) regions (including the 100-bp upstream and 100-bp downstream sequences) of 19,111 protein-coding genes or the exon regions of 20,268 non-coding genes and possessed low off-target scores (≤0.05) were selected and combined with the Brunello, GecKOv2 AB, Sabatini, TorontoKoV3, and YusaKoV1 libraries after excluding sequences containing *Bsm*BI and *Aar*I restriction enzyme cleavage sites. Each target sequence included a 23-nt PAM-endowed sequence and a 40-nt context extending 20 nt in the 5′ and 3′ directions of the endogenous gRNA-encoding sequence. The AIdit_ON library, which contained 743,344 gRNAs paired with their corresponding targets, was independently synthesized in the form of 8 oligonucleotide pools (GeneScript) in which each oligonucleotide contained a *Bsm*BI restriction site, a 20-nt gRNA-encoding sequence, two inward-directed *Aar*I restriction sites (for cloning the gRNA scaffold), a 63-nt putative genomic target sequence and a second *Bsm*BI site with a total length of 149 nucleotides.

To assemble the library for predicting off-target effects (Aldit_OFF), 1019 gRNA sequences from our on-target experiments were randomly selected for inclusion, resulting in a moderately dense distribution of indel efficiencies and the mononucleotide contexts of these sequences (Supplementary Fig. S21a, b). In the comprehensive analysis of off-target effects, we considered the numbers, positions and types of mismatches between the sgRNAs and target sequences and the effects of DNA/RNA bulge types and their positions on the off-target activities of SpCas9. Specifically, the Aldit_OFF library consisted of the following randomly selected components. (1) For the single mutation type, a total of 88,711 pairs of gRNAs and target sequences were designed, comprising 1019 gRNAs * 69 (= 23 positions * 3 (all possible substituted bases)) for the single mismatch type, 500 gRNAs * 21 (deletion positions from 1 to 21) for the single deletion type and 100 gRNAs * 79 (= 4 insertion bases * 19 insertion positions from 2 to 20 + 3 insertion bases without G * 1 (insertion position of 21)) for the single insertion type. After deduplication and data supplementary, 85,039 oligonucleotide pairs of gRNAs and target sequences were synthesized. (2) For the multiple mismatch type, we adopted two different strategies for selecting pairs of gRNAs and target sequences: the traversal strategy and whole-genome searching. Under the traversal strategy, we first split the target region into 5 windows with overlap occurring every 7 positions (the window positions ranged from 1 to 7, from 5 to 11, from 8 to 14, from 12 to 18, and from 15 to 21) and selected 58,500 multi-mismatches of up to six nucleotides, including 100 randomly selected gRNAs * 5 windows * 119 multiple-position combinations from 2 to 6 for each window * 1 substituted base combination with the highest CFD score. In addition, we randomly selected 100 gRNAs, searched for potential off-target sites of these gRNAs in the genome using the Elevation-search tool and thereby obtained 35,079 pairs of gRNAs and target sequences with up to 6 multi-mismatches, giving priority to high CFD scores. After data deduplication and supplementary, 94,579 oligonucleotide pairs of gRNAs and target sequences were synthesized. (3) To evaluate off-target effect quantification performance achieved via the high-throughput methods, the Aldit_OFF library also contained 431 off-target sites of 13 gRNAs measured with GUIDE-seq or Digenome-seq^27,35–40^ and 805 pairs of gRNAs and target sequences (of 3 gRNAs) evaluated based on a similar oligonucleotide synthesis method^32^. We also added 1035 matched sequences for all gRNAs to the Aldit_OFF library as references and selected 1243 same off-targets between two independent AIdit_OFF libraries as inernal reference (Supplementary Table S5).

### Plasmid design and construction

ON-OFF-Backbone_lentiGuide-Puro-Mkate2: Two inward-directed *Bsm*BI restriction sites, a fixed 28-bp nontarget sequence, and two inward-directed *Aar*I restriction sites were synthesized and cloned into the 3′ end of the U6 promoter of Direct-seq_lentiGuide-Puro-Tail-8A8G (Addgene #52963). *Bsm*BI restriction sites for cloning the gRNA-target library inserts were located downstream from the U6 promoter, and *Aar*I restriction sites were used for the cloning of the BC. The resulting plasmid was referred to as ON-OFF-Backbone_lentiGuide-Puro-Mkate2.

ON-OFF-Insert-Scaffold: 2 *Aar*I restriction sites, 1 conventional gRNA scaffold sequence, and 7 consecutive thymines (T) were synthesized and cloned into pET-28a(+), resulting in the ON-OFF-Insert-Scaffold vector (kanamycin resistance marker).

### lenti-Cas9-Blast: lenti-Cas9-Blast (Addgene #52962)

lenti-DNTT-HygR: The human *DNTT* CDS was PCR amplified from Jurkat cell cDNA using the *DNTT*-F/*DNTT*-R primers. The hygromycin resistance gene was PCR amplified from a plasmid containing the gene (Addgene #89308). The two PCR products were inserted into the *Bsi*WI and *Eco*RI sites of our lentivirus vector by Gibson assembly, resulting in the plasmid vector lenti-DNTT-HygR.

The corresponding all-in-one plasmids used for measuring SpCas9 indel frequencies at endogenous sites were constructed by inserting a gRNA oligo into an empty backbone (Addgene #82416) via Golden Gate assembly.

The gRNA plasmid employed for *DNTT* knockout (*DNTT*-KO) was constructed by inserting a gRNA oligo into our empty gRNA backbone via Golden Gate assembly. The gRNAs were designed by using the AIdit_Cas9_ON model developed in this study. The primer sequences used to construct the vectors and gRNA sequences are listed in Supplementary Table S7.

### Plasmid library preparation

Plasmid library preparation involved a three-step Golden Gate cloning process. *Step 1*: A BC oligo (including a 20-nt random nucleotide flanked by two outward-directed *Aar*I restriction sites) was synthesized by GenScript. Fwd-Barcode/Rev-Barcode primers were used to amplify the BC oligo using 25 µL of NEBNext Ultra II Q5 Master Mix (New England Biolabs), 1 µL of BC oligo (~0.5 ng), 5 µL of primer mix (final concentration 500 nM), and 19 µL of water. The following PCR program was applied: i. 98 °C for 30 s. ii. 8 cycles of (98 °C for 10 s; 67 °C for 30 s; 72 °C for 10 s). iii. 72 °C 2 min. iv. 4 °C hold.

The 8 PCR products were combined and purified using a QIAquick Nucleotide Removal Kit (QIAGEN #28306). The purified total product was then cloned into the ON-OFF-Backbone_lentiGuide-Puro-Mkate2 plasmid via Golden Gate assembly (90 cycles of 5 min at 37 °C and 5 min at 22 °C, followed by a 30-min heat inactivation at 65 °C) using *Aar*I and purified with Agencourt AMPure XP SPRI beads (XP beads) according to the manufacturer’s instructions. The purified plasmid library was transformed into Endura (Lucigen) electrocompetent cells, which were subsequently grown at 30 °C for 20 h on Luria-Bertani (LB) agar plates with 100 µg/mL ampicillin (a limited dilution series was also plated and grown to assess transformation efficiency). To achieve high coverage, more than 1 × 10^8^ colonies (>1000×) were scraped, and plasmid DNA was extracted using a Plasmid Midiprep kit (Qiagen). The resulting plasmid library was referred to as Lig1-ON-OFF-Barcode. *Step 2*: The 10 oligonucleotide pools were synthesized by GenScript, and each oligonucleotide pool was PCR- amplified using the Fwd-oligo-pool/Rev-oligo-pool primers in the following PCR mixture: 25 µL of NEBNext Ultra II Q5 Master Mix (New England Biolabs), 2 µL of the oligonucleotide pool (~40 ng), 5 µL of the primer mix (final concentration 500 nM), and 18 µL of water. The following PCR program was applied: i. 98 °C for 30 s. ii. 18 cycles of (98 °C for 10 s; 60 °C for 30 s; 72 °C for 10 s). iii. 72 °C for 2 min. iv. 4 °C hold.

We performed 24 separate amplification reactions in 50-µL PCR mixtures for every oligonucleotide pool, and the total PCR product from the 24 combined PCR products was concentrated to 200 µL using Amicon Ultra 0.5-mL Centrifugal Filters (Sigma). The concentrated PCR product was gel purified (2.5% agarose gel, 120 V for 10 min, 80–100 V for 60 min) using a QIAquick Gel Extraction Kit, and the gel-purified product was further purified via phenol–chloroform extraction and ethanol precipitation. This purified product was cloned into the Lig1-ON-OFF-Barcode plasmid via Golden Gate assembly using *Bsm*BI. The remaining library preparation procedures performed in Step 2 were essentially the same as those in Step 1, and the resulting plasmid library was referred to as Lig2-ON-OFF-Barcode-Oligo_pool (Coverage > 1000×). *Step 3*: The ON-OFF-Insert-Scaffold was cloned into Lig2-ON-OFF-Barcode-Oligo_pool via Golden Gate assembly using *Aar*I and purified with XP beads. The purified plasmid library was transformed into Endura (Lucigen) electrocompetent cells. A limited dilution series was plated and grown to assess transformation efficiency. The total colonies grown on LB agar plates (>1000×) were subsequently scraped, and plasmid DNA was extracted using a Plasmid Midiprep kit (Qiagen). Accordingly, we obtained a final plasmid library coverage of 300× relative to the initial number of oligonucleotides. All 3 steps of plasmid library preparation were repeated in exactly the same way for each oligonucleotide pool. Biological replicates for each oligonucleotide pool were performed using two independent plasmid library preparations. The primer sequences used to construct the plasmid library are listed in Supplementary Table S7.

### Cell culture and cell line establishment

The HEK293T cells were a gift from the Shang Cai lab at Westlake University. The K562 cells were purchased from the American Type Culture Collection (ATCC). The Jurkat cells were a gift from the Xu Li lab at Westlake University. The H1 cells were obtained from the National Collection of Authenticated Cell Cultures. The HEK293T cells were grown in high-glucose Dulbecco’s modified Eagle’s medium (DMEM) supplemented with 10% fetal bovine serum (FBS) (Gemini #900-108) and 1% penicillin/streptomycin (Gibco #15140-122). K562 and Jurkat cells were grown in RPMI 1640 (SIGMA #R8758) with 10% FBS (Gemini #900-108) and 1% penicillin/streptomycin. H1 cells were cultured in Matrigel (Corning)-coated plates with mTeSR medium (StemCell Technologies). The medium was changed daily. The cells were passaged every 3–4 days using ReLeSR (StemCell Technologies). The K562-Cas9 cell line was established by inserting the lenti-Cas9-Blast (Addgene #52962) lentivirus into WT K562 cells. A single clone was selected, expanded, and maintained with 2 μg/mL blasticidin. The Jurkat-Cas9 cell line was established following a similar procedure^41^. To establish the *DNTT*-KO Jurkat-Cas9 cell line, electroporation was used to transiently transfer the *DNTT*-KO gRNA plasmid into Jurkat-Cas9 cells. Single Mkate2-positive cells were isolated via fluorescence-activated cell sorting (FACS) and replated to generate a monoclonal lineage at 48 h post-transfection. A small portion of the cells were assessed to verify the KO of the DNTT protein. The *DNTT*-OE K562-Cas9 cell line was established by inserting the lenti-DNTT-HygR lentivirus into K562-Cas9 cells. A single clone was selected, expanded, and maintained with 100 μg/mL hygromycin. A small fraction of the cells were selected to confirm stable *DNTT* gene expression.

### Lentivirus production and transduction of human cells

For lentivirus production, the transfer plasmids containing the GOI or the plasmid library, psPAX2 and pMD2.G were combined at a weight ratio of 5:3:2 to yield a plasmid mixture of 192 µg, which was then delivered in an equal volume to 90% confluent HEK293T cells cultured in 2 T175 flasks using the calcium phosphate transfection method according to the manufacturer’s protocol^42,43^. At 6–8 h after transfection, the cells were washed twice with PBS (20 mL) to remove residual plasmids, and the PBS was then replaced with 20 mL of fresh Advanced DMEM (Gibco) supplemented with 2% FBS. The supernatant containing the virus was collected at 48 h and 72 h after transfection and filtered through a 0.45-μm polyvinylidene fluoride filter. After ultracentrifugation of the supernatant (20,000 rpm for 2 h), the virus was resuspended in 1 mL of PBS and stored at −80 °C in small aliquots. To determine the virus titer, the viral aliquots were serially diluted and transduced into corresponding suspension cell lines in the presence of 8 µg/mL polybrene (Sigma); this was followed by centrifugation at 600× *g* for 2 h at 32 °C. For adherent cell lines, the viruses were transduced into corresponding cell lines seeded on day 1, to which 8 µg/mL polybrene was added on day 2. After incubating the cells for 48 h at 37 °C under standard cell culture conditions, the ratio of Mkate2-positive cells was analyzed by FACS. The titer was calculated according to the following formula developed by Jakob Reiser^44^: IU ml^−1^ = (*F* × *N* × *D* × 1000)/*V*, where *F* = the percentage of fluorescent cells, *N* = the number of cells at the time of transduction, *D* = the fold dilution of the vector sample used for transduction, and *V* = the volume (µL) of the diluted vector sample added to each well for transduction. We calculated the titer only if the percentage of fluorescent cells was less than 25%.

### Screening and sequencing

For the screening of the Jurkat-Cas9 and K562-Cas9 cell lines using the AIdit_ON and Aldit_OFF libraries, 15 million cells were resuspended in 6 mL of RPMI in the presence of 8 µg/mL polybrene, and we then transferred 1 mL of the cell suspension to each well of a 6-well plate. A predetermined volume of virus in the same 6-well format was added to each well to achieve an MOI of 5. After centrifugation at 600× *g* for 2 h at 32 °C, 2 mL of prewarmed RPMI with 15% FBS and 8 µg/mL polybrene was immediately added to each well of the 6-well plate. The cells were selected with puromycin (2.5–3 µg/mL) for 2 days beginning at 36 h post-transduction to remove uninfected cells. The cells were then harvested at day 3.5 after transduction, and genomic DNA (gDNA) was isolated using DNeasy Blood & Tissue Kits (Qiagen) according to the manufacturer’s protocol. The region that was integrated with gDNA, including the gRNA, target sequence, and barcode, was PCR-amplified from gDNA for HTS. The Fwd-libseq/Rev-libseq primers for HTS were used to specifically amplify all of the gDNA using 25 µL of NEBNext Ultra II Q5 Master Mix (New England Biolabs), 17 µL of gDNA (~2.5 µg), 2.5 µL of primer mix at a final concentration of 250 nM, and 5.5 µL of water. For each gDNA sample, the number of amplification reactions ranged from 30 to 60. The following PCR program was applied: i. 98 °C for 30 s. ii. 10 cycles of *(98 °C for 10 s; 64 °C for 30 s; and 72 °C for 10 s) + a variable number of cycles (8 to 13) of *(98 °C for 10 s; 71 °C for 30 s; and 72 °C for 10 s). iii. 72 °C for 2 min. iv. 4 °C hold.

The Fwd-libseq/Rev-libseq primers were also used to amplify the final AIdit_ON and Aldit_OFF plasmid libraries using 25 µL of NEBNext Ultra II Q5 Master Mix (New England Biolabs), 1 µL of the plasmid library (~40 ng), 2.5 µL of primer mix at a final concentration of 250 nM, and 21.5 µL of water. For each plasmid library, eight PCR replicates were performed. The following PCR program was applied: i. 98 °C for 30 s. ii. 6 cycles of *(98 °C for 10 s; 64 °C for 30 s; and 72 °C for 10 s) + 8 cycles of *(98 °C for 10 s; 71 °C for 30 s; and 72 °C for 10 s). iii. 72 °C for 2 min. iv. 4 °C hold.

The PCR products were purified with XP beads and quantified and sequenced on an Illumina NovaSeq system via 150-bp paired-end sequencing. If a mutation was found in the gRNA sequences or gRNA-target sequences in the final plasmid libraries compared with the library sequences we originally designed, the corresponding gRNA was excluded from further analysis because of the error derived from oligonucleotide synthesis or PCR amplification. All screening and sequencing steps were repeated for each oligonucleotide pool. We performed two biological replicates of each oligonucleotide pool. The primer sequences used in these steps are listed in Supplementary Table S7.

### Detection of SpCas9 indel frequencies at endogenous sites and GUIDE-seq

To obtain the SpCas9-Endo dataset, 96 gRNA sequences from human endogenous sites were randomly selected. The corresponding all-in-one plasmids containing SpCas9, gRNA, and GFP were generated via Golden Gate assembly. HEK293T cells were plated at a density of 3 × 10^5^ per well in 2 mL of media in poly-*D*-lysine-coated 6-well plates. After 24 h, the cells typically reached 90% confluence, and the plasmids (2.5 μg) were then transfected with Lipofectamine 3000 (Invitrogen #L3000-015) according to the manufacturer’s instructions. For K562 and H1 cells, an easy-to-perform and low-cost method for our lab is using viral vectors to deliver the Cas9/sgRNA into the cells. For lentivirus production, the transfer plasmids containing the corresponding sgRNAs, psPAX2, and pMD2.G were combined at a weight ratio of 5:3:2 to yield a plasmid mixture of 25 µg, which was then delivered in 90% confluent HEK293T cells cultured in T25 flask using the calcium phosphate transfection method according to the manufacturer’s protocol. At 6–8 h after transfection, the cells were washed twice with PBS to remove residual plasmids, and the PBS was then replaced with 5 mL of fresh Advanced DMEM (Gibco) supplemented with 2% FBS. The supernatant containing the virus was collected at 48 h after transfection and filtered through a 0.45-μm polyvinylidene fluoride filter. The virus was repackaged in 1 mL and stored at −80 °C for further use. To transduction, the viral aliquots were transduced into K562 cell line in the presence of 8 µg/mL polybrene (Sigma); this was followed by centrifugation at 600× *g* for 2 h at 32 °C. For H1, the viruses were transduced into H1 cell line seeded on day 1, to which 8 µg/mL polybrene was added on day 2. The cells were selected with puromycin (2.5–3 µg/mL) for 2 days beginning at 36 h post-transfection to remove uninfected cells. The cells were then harvested at day 3.5 after transduction, and genomic DNA (gDNA) was isolated using DNeasy Blood & Tissue Kits (Qiagen) according to the manufacturer’s protocol. The region that sgRNA targeting was PCR amplified from gDNA for HTS for 96 sgRNAs, respectively.

For GUIDE-seq, 5 gRNA sequences from human endogenous sites were randomly selected. The corresponding all-in-one plasmids containing SpCas9, gRNA, and GFP were generated by Golden Gate assembly. When the HEK293T cells typically reached 90% confluence, 5 μg of the plasmids and 50 pmol of dsODN were transfected into the cells with Lipofectamine 3000 (Invitrogen #L3000-015). At 48 h post-transfection, the cells were lysed, and gDNA was extracted using a TIANamp Genomic DNA Kit (TIANGEN #DP304-03). The in vitro cleavage of genomic DNA and GUIDE-seq sequencing were carried out according to the manufacturer’s instructions.

### Analysis of indel frequencies

After performing deep sequencing, we analyzed the obtained sequencing reads using data processing procedures, comprising sequence alignment, read quality control, and indel frequency analysis based on in-house Python scripts. We defined the indel (insertions or deletions) window located around the cleavage site (i.e., the 7-nt region next to the upstream region of the PAM). First, through deep paired-end sequencing, the correspondence between each gRNA-target sequence and the BCs was determined in the plasmid library without genome editing. Then, we conducted sequence alignment between the designed gRNA sequences and sequencing reads in both the plasmid and edited libraries using Bowtie. To remove the background unmatched reads derived from the synthesis or PCR amplification procedures, we excluded these error reads from the edited library in the subsequent analysis.

Specifically, for the on-target case, we calculated the perfect matching rate with the designed sequences in the same gRNA-barcode sequencing reads in the plasmid library and filtered out the low-quality gRNA-barcode reads for which the matching rate was less than 90%. Then, we aligned and only retained the gRNA-barcode reads in the edited library corresponding to high-quality reads in the plasmid library and removed the background errors originating from the gRNA-barcodes of the plasmid library. Thus, the indel frequency of a gRNA was calculated with the following formula:

$${\rm{Indel}}\,{\rm{frequency}} = \frac{{\mathop {\sum}\nolimits_{{\rm{barcode}}\,{\rm{per}}\,{\rm{gRNA}}} {{\rm{Number}}\,{\rm{of}}\,{\rm{indel}}\,{\rm{reads}}} }}{{\mathop {\sum}\nolimits_{{\rm{barcode}}\,{\rm{per}}\,{\rm{gRNA}}} {{\rm{Number}}\,{\rm{of}}\,{\rm{total}}\,{\rm{reads}}} }}.$$

To accurately measure the editing efficiencies of off-targets, after sequence alignment, firstly we classified each sequence read according to its gRNA-target-barcode in plasmid library and calculated ratio of the dominant type in each category, and filtered out low-quality categories which dominant ratio is less than 90%. Then for the edited library, we classified and retained sequence reads of the library based on high-quality gRNA-target-barcode categories of plasmid library. To remove background errors from the synthesis or PCR procedure of the plasmid library, we filtered out gRNA-target-barcode reads of the edited library same with non-dominant ones in the corresponding category of the plasmid. And for the synthesis or PCR errors from the edited library, if the mutated result of a sequence read in the library was not located in the edited window and did not include the dominant type of the corresponding category of the plasmid, then we identified and remove the read as a background error from the edited library. Thus, we can calculate the indel frequency of a gRNA-target from the off-target library as follows:

$${\rm{Indel}}\,{\rm{frequency}} = \frac{{\mathop {\sum}\nolimits_{{\rm{barcode}}\,{\rm{per}}\,{\rm{gRNA}}\_{\rm{target}}} {{\rm{Number}}\,{\rm{of}}\,{\rm{indel}}\,{\rm{reads}}} }}{{\mathop {\sum}\nolimits_{{\rm{barcode}}\,{\rm{per}}\,{\rm{gRNA}}\_{\rm{target}}} {{\rm{Number}}\,{\rm{of}}\,{\rm{total}}\,{\rm{reads}}} }}.$$

To correct the batch effects along with the experimental processing of the eight libraries, a Baseline Library was constructed to include 10,000 gRNAs randomly chosen from each of the library1 to library8. These gRNAs in the Baseline Library served as internal references of the eight subpools. Through fitting a linear regression model using scikit-learn between indel frequencies of shared gRNAs from each subpool and the Baseline Library, the indel frequencies of all gRNAs were normalized.

### Analysis of the SpCas9-induced DSB repair profile

As described above, we classified the edited reads of each gRNA into the DSB repair categories of deletion and insertion based on their types, locations, and sizes with respect to the edited window. Due to the difference between the SpCas9-induced DSB repair distributions of K562 and Jurkat cells, which showed that Jurkat cells preferentially exhibited the introduction of longer insertions than were observed in K562 cells, we grouped all insertion mutations into different insertion categories in K562 and Jurkat cells (see the corresponding portion of the text for details). We normalized all repair categories for each gRNA according to the total number of indel reads for that gRNA. To achieve prediction robustness, we merged adjacent categories, with one category whose mean frequency was less than 0.0001 and one whose Pearson coefficient between two biological replicates was less than 0.5. Finally, 117 repair categories were identified in K562 cells and 163 in Jurkat cells.

### Symmetrized KL divergence

KL divergence (or the relative entropy of probability distributions) was used to evaluate how the distribution of the measured SpCas9-induced DSB repair outcomes differed from that of the predicted repairs. To avoid the influence of the asymmetry of KL divergence, we used symmetrized KL divergence. The formula for calculating symmetrized KL divergence was as follows:

$$D_{{\rm{symmetrized}}\,{\rm{KL}}}\left( {P||Q} \right) = \mathop {\sum}\limits_{x \in \aleph } {\left\{ {P(x)\log \left( {\frac{{P(x)}}{{Q(x)}}} \right) + Q(x)\log \left( {\frac{{Q(x)}}{{P(x)}}} \right)} \right\}/2}$$

where *P* and *Q* are two discrete probability distributions of the measured and predicted DSB repair outcomes per gRNA, respectively, and ℵ is a set of DSB repair categories that we defined. We added a 0.00001 epsilon value to each element of *P* and *Q* in the computational code such that neither *P*(*x*) nor *Q*(*x*) was equal to 0, where *x* is one repair category in ℵ.

### Feature engineering

To evaluate the effects of the input sequences on SpCas9 on-target activities, we used sequence features of different lengths as the model inputs. These included the only 23-bp sequence (20-bp target sequence and 3-bp PAMs); 24-bp to 28-bp sequences obtained by the addition of 1-bp to 5-bp nucleotides to the upstream and downstream regions of the 23-bp sequence; and 33-bp, 43-bp, 53-bp, and 63-bp sequences obtained by adding 5-bp, 10-bp, 15-bp, and 20-bp nucleotides, respectively, to the upstream and downstream regions of the 23-bp sequence. Finally, we used 63-bp (20-bp upstream + 20-bp target + 3-bp PAM + 20-bp downstream) one-hot sequence encoding as input of AIdit_ON.

For the development of off-target models, we extracted pairs of gRNAs and target sequences to engineer features. We adopted a combination of multiple feature sets, which included the basic sequence features of gRNAs and targets, the aligned sequence features of gRNAs and target sequences, mismatch positions, the PAM nucleotides in the target sequences, the numbers of mismatches and the prediction features of the on-target model for sgRNA sequences and target sequences for which the upstream and downstream information was the same. The sequence features consisted of a 20-bp protospacer/potential target region and a 3-bp PAM. We trained and selected the optimal feature combination to train the models that produced both the highest validation scores across multiple metrics, including the Pearson correlation coefficients and Spearman correlation coefficients, and the highest MSEs between the experimentally measured and predicted gRNA off-target activities. We finally selected the best feature combination as input features of AIdit_OFF, which included 23-bp sequence features of both gRNAs and targets, position-dependent substitution types, the PAM types of targets, mismatch number and prediction values of AIdit_ON for both matched and unmatched target sequences. As we designed off-target sequences for each gRNA to reflect distinct sequences where off-target, there are multiple gRNA-off-target pairs corresponding to a single gRNA. Accordingly, we split these gRNA-off-target pairs into three subsets, in which gRNAs were mutually exclusive from each other to avoid data leakage. Specifically, the three subsets include a training set (741 gRNAs and 76,498 pairs), a validation set (93 gRNAs and 12,285 pairs), and an independent test set (92 gRNAs and 13,280 pairs).

To predict SpCas9-induced DSB repair outcomes, in addition to the sequence features used to train the models, we incorporated microhomology information into our models. The left-end nucleotides of each deletion were compared with the left-end nucleotides downstream of the deletion within the same length (1 bp to 4 bp, since each deletion was left-aligned). This yielded 2390 binary microhomology features by one-hot encoding for each 85-bp target sequence, including a 20-bp upstream component, a 20-bp protospacer, a 3-bp PAM, and a 42-bp downstream component. To investigate the factors that affected insertion or deletion in the repair outcomes, we exploited different feature sets to predict the distribution of insertions or deletions alone. Similar to the findings of a previous study^28^, the results showed good performance (Pearson correlation coefficient median: 0.977, Supplementary Fig. S22) only for the sequence features around the cut site (+/–3 bp) used to train the insertion model, and the addition of longer sequences or microhomology features slightly improved performance in predicting the insertion distribution. However, when predicting the deletion distribution, the microhomology features played a key role. The median Pearson correlation coefficients were 0.644, 0.892 and 0.905 (Supplementary Fig. S22) for only sequence features, only microhomology features and the combination thereof, respectively. We trained 117 and 163 XGBoosts for each category of DSB repair outcomes using both the sequence features and microhomology features of K562 and Jurkat cells, respectively. Finally, AIdit_DSB output 117 or 163 observed repair categories for K562 or Jurkat (supplemented in Fig. 3D) since there are lots of DSB-induced repair outcomes which are merged because of low ratio or poor repeatability between two biological replicates. Final feature sets for training the AIdit_DSB include the 63-bp sequence, 2390 microhomology features and prediction values of corresponding XGBoost for cell lines.

### Hyperparameter optimization

The Python Hyperopt optimization library was developed to search optimized hyperparameter combinations of models from the configuration space based on Bayesian optimization algorithms. The search space includes both real-valued and discrete-valued dimensions. We optimized the hyperparameter combinations of our models through multiple iterations of the hyperparameter space. First, we identified the initial hyperparameter search space based on the performance of the models with several random parameter combinations and then used Hyperopt to perform distributed hyperparameter optimization to reduce and optimize the hyperparameter space based on the tree-structured Parzen estimator approach (TPE). Finally, we selected the optimized hyperparameter combinations of the models based on their validation scores. Notably, when training the on-target models, we randomly sampled 10,000 gRNA data from the training dataset to reduce the time required for model selection.

### Development of deep learning-based models

CNNs and RNNs are two powerful types of deep learning-based algorithms that have recently been applied to predict SpCas9 activities and promoter–enhancer interactions and the optimization and design of diverse proteins.

Our CNN architecture for predicting gRNA activities consisted of an input layer, convolution layers, pooling layers, a flattening layer, fully connected layers, and an output layer. The convolution layers calculated a dot product between the convolution kernel and the input layer’s matrix to learn spatially local correlations. The max pooling layers were used to reduce the dimensions of the data from the convolution layer and output feature maps. We used a flattening layer to flatten the feature map to transform the data format to the shape of (batch size, flattened neuron number), which was input into the fully connected layers. Similar to an MLP, fully connected layers connect every neuron in the input to every neuron in the next layer via a weighted sum operation and a rectified linear unit (ReLU) nonlinear function and finally output the solution. During CNN architecture building, we selected two interchangeable convolution and pooling layers and two fully connected layers.

When training the CNNs, the input sequence data were converted into four-dimensional binary matrix data via one-hot encoding. Hyperparameters including the number of filters, kernel sizes, the number of neurons in the hidden layers and batch size were optimized by using Hyperopt based on the following configurations: the number of filters was chosen from [32, 64, 128, 256, 512], kernel size was selected from [2, 3, 4], the number of neurons in the hidden layers was chosen from [64, 128, 256], and batch size was selected from [128, 256, 512, 1024]. To reduce model overfitting, we added a dropout layer after the first fully connected layer with a dropout rate of 0.3. When training the CNNs, we performed early stopping by monitoring the MSE of the validation dataset, where training was stopped once the metric stopped decreasing after 50 iterations.

A long short-term memory network (LSTM) is a powerful artificial RNN architecture for processing the sequences of patterns. An LSTM unit is a cell including an input gate, a forget gate, and an output gate, which plays a key role in remembering information for long periods of time and avoiding the long-term dependency problem. Since LSTM networks process time series data, when 63-bp sequences were input, we converted the sequences into the shape of a (63, 4) binary matrix via one-hot encoding, indicating that the input data of each batch had 63 time steps and that, at each time step, the input was a 4-dimensional vector. For time step *t*, let *x*_*t*_, *h*_*t*−1_, and *c*_*t*−1_ be the input 4-dimensional vector, the hidden state vector at time step *t*−1 and the cell state vector at time step *t*−1, respectively. The following equations represent the progression of the information flow in the inner LSTM unit:

$$f_t = \sigma (W_fx_t + U_fh_{t - 1} + b_f)$$

$$i_t = \sigma (W_ix_t + U_ih_{t - 1} + b_i)$$

$$o_t = \sigma (W_ox_t + U_oh_{t - 1} + b_o)$$

$$c_t = f_t \ast c_{t - 1} + i_t \ast \tanh (W_c \ast x_t + U_c \ast h_{t - 1} + b_c)$$

$$h_t = o_t \ast \tanh (c_t)$$

where W ∈ R^h×63^, U ∈ R^h×h^, and b ∈ R^h^ are weight matrices and trainable bias vector parameters, and *σ*, tanh and * are sigmoid functions, hyperbolic tangent functions and elementwise multiplication functions, respectively. *f*_*t*_, *i*_*t*_, and *o*_*t*_ are the forget, input, and output of gates at time step *t*, respectively, and *c*_*t*_ and *h*_*t*_ are the cell state and hidden state vector at time step *t*−1.

We used bidirectional LSTM (BiLSTM) networks to learn the input sequence once from the 5′ to 3′ direction and once from 3′ to 5′ direction during every time step. The two output sequences from the BiLSTM networks were combined by using a concatenation function.

The LSTM-based model architecture that we developed included layers consisting of BiLSTM units, fully connected layers and an output layer. We used dropout layers in both the BiLSTM layers and fully connected layers to prevent overfitting, and we used ReLU activation functions to increase the nonlinear properties of the models following the fully connected layers. The Hyperopt strategy was used to select the optimized hyperparameters, including the numbers of hidden neurons in the BiLSTM and fully connected layers, dropout rates, and batch size. Similar to CNN model training, the early stopping strategy was adopted to increase the training efficiency by stopping training when the MSE of the validation set failed to improve further during 50 consecutive iterations. In addition, we exploited a learning rate schedule to reduce the learning rate by half every 50 epochs, from the initial value of 0.001 to the minimal value of 1e–5.

In the training of the CNN and RNN models, the metrics that we used were the MSE and Spearman correlation score, the loss function was the MSE function, and an adaptive moment estimation (Adam) optimizer was used for stochastic gradient descent (SGD). After selecting the optimized hyperparameter combination, tenfold cross-validation was performed to evaluate the generalization performance of the models. The final models were trained using our corresponding combined training and validation dataset for K562 and Jurkat cells. The development of the deep learning-based models was implemented using Keras built on top of TensorFlow.

### Development of conventional machine learning-based models

Regardless of whether we were performing on-target or off-target modeling, we trained multiple conventional machine learning-based algorithms separately using scikit-learn (version 0.21.1); this training was applied to ridge linear regression, lasso linear regression, elastic linear regression, random forest, GBDT, XGBoost, and MLP algorithms. After engineering the features as previously described for on- and off-targets, we selected the optimized parameter combination of each model listed above via the Hyperopt method over at least 50 max iterations based on the following searching spaces: for linear regression, the L1-regularized parameter was chosen from 1e–4 to 150, and the L2-regularized parameter was selected from 1e–9 to 0.01; for the algorithms based on decision trees, the n_estimators parameter ranged from 50 to 2000, max_depth ranged from 6 to 50, min_samples_split ranged from 50 to 500, min_samples_leaf ranged from 30 to 200 and max_features ranged from 0.5 to 1.0; for the MLP, the selected sizes of multiple hidden layers ranged from 30 to 650, the L2 penalty parameter ranged from 1e–5 to 0.1, and the maximum number of iterations ranged from 50 to 500.

For DSB repair modeling, we used the ensemble strategy to predict DSB repair outcomes. As previously described, the prediction of each category by XGBoost involved inputting both sequence and microhomology features into a multiple-category logistic regression model as the final training features; this process was implemented using TensorFlow. To select the optimized parameters of the logistic regression model, we used a grid search based on the following parameter configuration: the optimization algorithm for the models was chosen from [SGD, Adam], the learning rate was chosen from [0.1, 0.01] and the batch size was selected from [1024, 512, 256, 128, 64].

### Public data collection and model comparisons

For on-target, the Spearman correlations between the experimentally measured sgRNA activities and the scores predicted by each on-target model were used to evaluate the performance of the models. To systematically compare the performance of AIdit_ON with that of the other existing models, we used 10 published endogenous datasets collected by Haeussler et al.^21^ from other groups. Each of these datasets included more than 100 gRNAs and was generated using the U6 promoter^1,2,5,13,16,6,18–20^. When evaluating these test datasets, we excluded the gRNA sequences included in our training datasets and did not compare the results with the correlations that were generated by models tested against their own training datasets to prevent bias from algorithmic overfitting.

For off-target, we compared the performance of AIdit_OFF with that of other conventional machine learning-based models trained on our high-throughput dataset using multiple metrics, including the Pearson and Spearman correlation coefficients and MSE between the measured off-target activities and the prediction scores yielded by each model. To evaluate the prediction accuracy of AIdit_OFF in quantifying real off-target effects, we used four independent datasets obtained from off-target unbiased genome-wide assays, including three GUIDE-seq datasets from Tsai et al.^35^, Kleinstiver et al.^33^, and Listgarten et al.^34^ and one dataset collected by Haeussler et al.^21^. We took advantage of the Elevation-search tool^34^ to perform efficient genomic searches for potential off-targets with the following search parameters: mismatchTolerance of six and mismatchLimit of 999,999,999, meaning that the possible off-target sites that we searched included those with a maximum of six nucleotide mismatches across 1–20 nucleotides of one gRNA, without a limit regarding the number of potential off-target sites for each gRNA. For the PAM selection of potential off-target sites, Elevation-search only considered nonzero active PAMs according to the CFD model (i.e., NAG, NCG, NGA, NGC, NGG, NGT, and NTG). Thus, 10, 9, and 23 gRNAs were included from the Tsai et al.^35^, Kleinstiver et al.^33^, and Listgarten et al. datasets^34^, respectively, which yielded 386, 228, and 62 active off-target sites based on GUIDE-seq and led to the identification of 963,321, 792,711, and 1,207,002 potential off-target pairs by Elevation-search. For the Haeussler data^21^, which were collected from eight different studies in which real off-target gRNA sites were identified through techniques such as targeted sequencing, GUIDE-seq, HTGTS, DiGenome-seq, and BLESS^35–40^, we filtered out the off-targets detected by targeted sequencing since the sensitivity of the targeted PCR was far higher than that of the whole-genome assays. The remaining 17 gRNAs from the Haeussler dataset^21^ generated 494 active off-target sites from 1,698,400 possible off-target loci searched by Elevation-search^34^. We calculated CCTop and Hsu-Zhang scores based on the descriptions in the corresponding papers^38,45^. For the CFD score^6^, we implemented the approach of Haeussler et al.^21^ When we compared our method to these approaches, we excluded the training gRNAs in our test datasets and only evaluated potential off-target sites with NRG PAMs that accommodated CCTop.

For SpCas9-induced DSB repair models, we compared our AIdit_DSB model to two other existing high-performance models (ForeCasT and Lindel) based on our independent test dataset, the ForeCasT-Lindel combined test dataset and other datasets collected from cell lines, including REP1, CHO, E14TG2A, and HAP1cells^27,28^. Each indel of DSB repair profiles predicted by both Lindel and ForeCasT was characterized by its type, size, and location with respect to the response edited window, though the identifiers defined by Lindel and ForeCasT were different. To conduct an easy comparison between the models, we unified the DSB repair categories predicted by both Lindel and ForeCasT to the format that we characterized (631 repair categories including 610 deletions of up to 30 bp, 20 insertions of up to 2 bp and one insertion of more than 2 bp). For each gRNA, we used multiple metrics to evaluate the prediction performance of each model; these metrics included the Pearson correlation, symmetrized KL divergence, and MSE between the measured DSB repair distribution and the predicted outcomes produced by the model. For a fair comparison, we evaluated the performance of models based on the same merged repair categories (i.e., 117 repair categories were identified in K562 cells and 163 in Jurkat cells), which were achieved by using the corresponding merging tables of K562 and Jurkat (Supplementary Tables S3, S4), respectively.

### Evaluation of feature importance

Feature coefficients of the elastic regression model for predicting off-target indel efficiencies were used to analyze the effect of mismatched nucleotide position on off-target activities. We first calculated the variation coefficient of regression coefficients of each position in off-target sequences and then remeasured the position feature importance score as an integrated value by multiplying the maximum absolute value of the regression coefficients of each position by the coefficient of variation of the corresponding position. The results show that the larger the integrated value of one position is, the more important the position is for off-target activities.

### Statistical analyses

We used Steiger’s test to compare the Spearman correlation between the prediction scores obtained with AIdit_ON model (Fig. 2d, e) and one-way analysis of variance (ANOVA) followed by Tukey’s post hoc test to conduct pairwise comparisons between the SpCas9-induced DSB repair outcome predictions of multiple models (Fig. 3e). Statistical significance was calculated based on the corresponding formulas using the Python scipy.stats library.

## Supplementary information

Supplementary Fig merged

Supplementary Figure Legend

Supplementary Table S1

Supplementary Table S2

Supplementary Table S3

Supplementary Table S4

Supplementary Table S5

Supplementary Table S6

Supplementary Table S7

## Data Availability

All scripts and software code used in the study are available from our GitHub repository (https://github.com/Aidit-Models/CRISPR-AIdit.git/). In addition, the graphical user interface (GUI) is available at https://crispr-aidit.com for bioinformatic novices to harness these tools. We have deposited the raw sequencing data in the Gene Expression Omnibus (GEO) with accession number GSE181774.
