# Supplementary material for: Deep sampling of gRNA in the human genome and deep-learning-informed prediction of gRNA activities
Source: Cell Discov. 2023 May 16;9:48. doi: 10.1038/s41421-023-00549-9 (PMC10188485; doi:10.1038/s41421-023-00549-9)

Supplementary Fig. S1

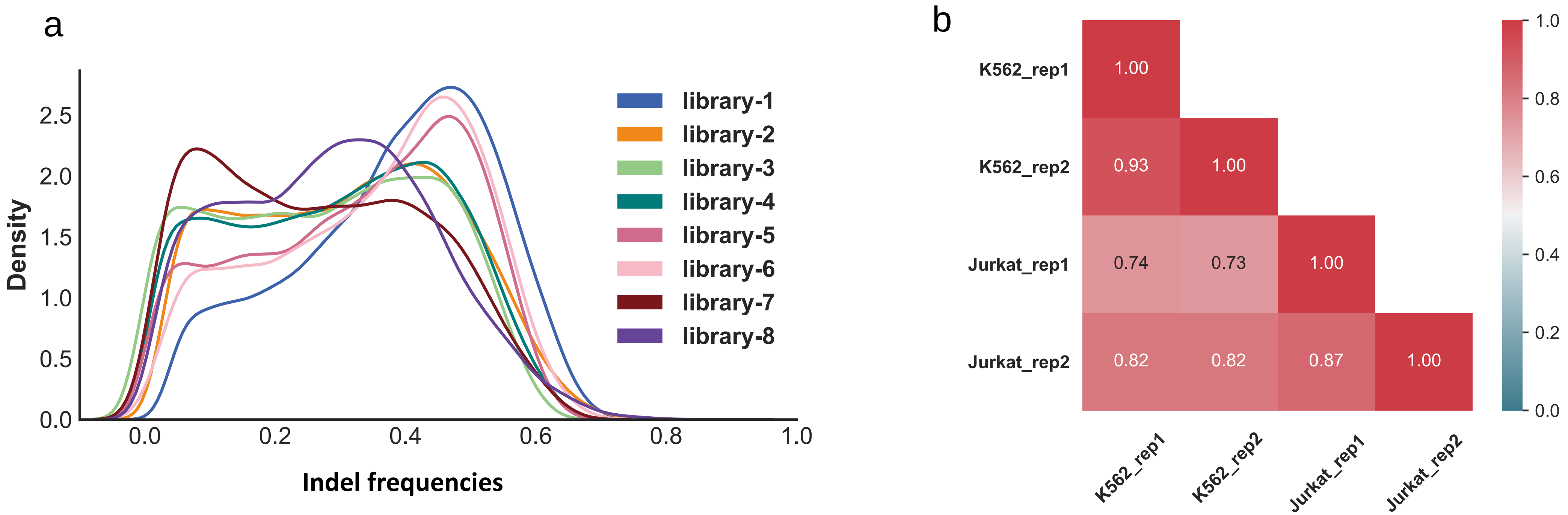

Supplementary Fig. S2

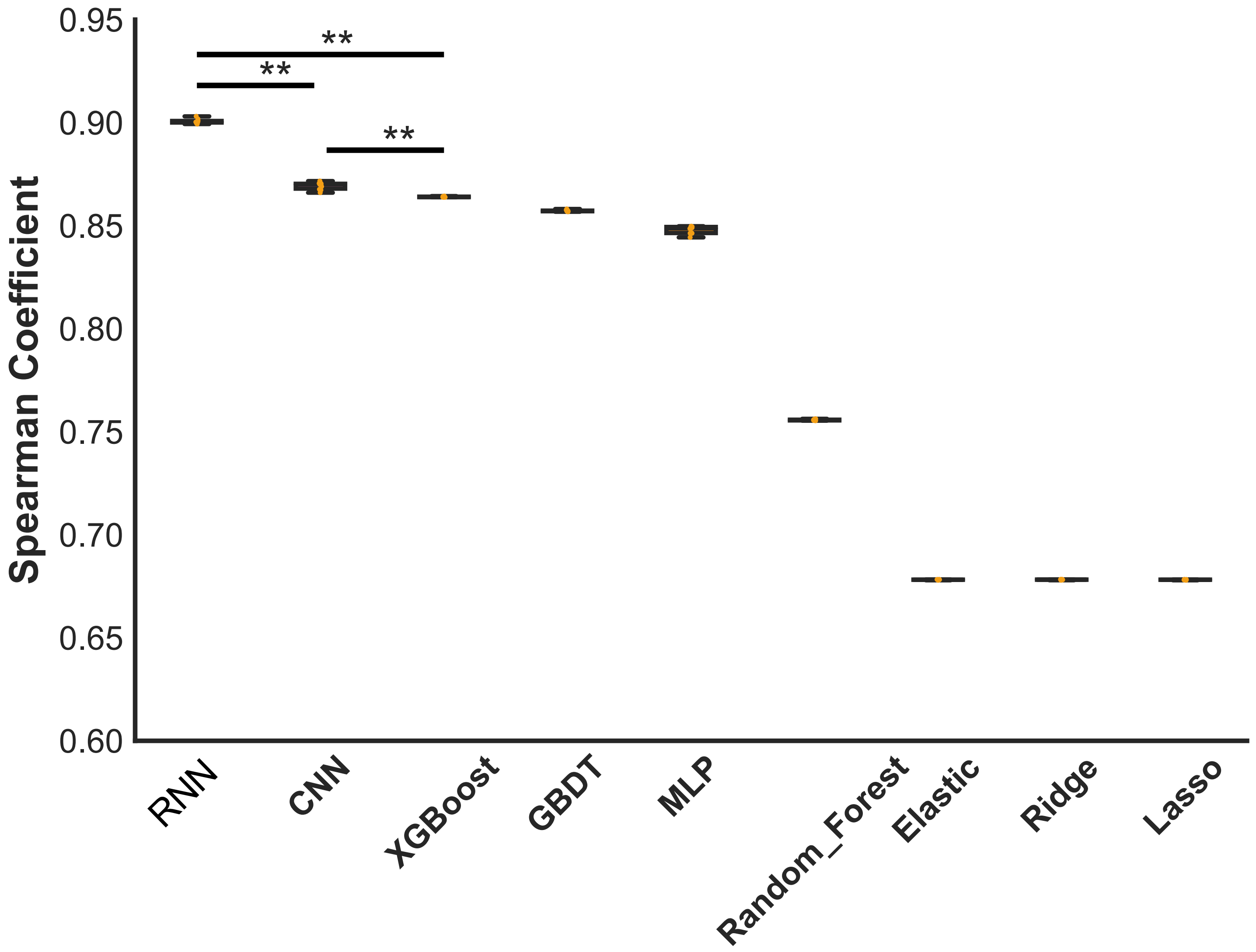

Supplementary Fig. S3

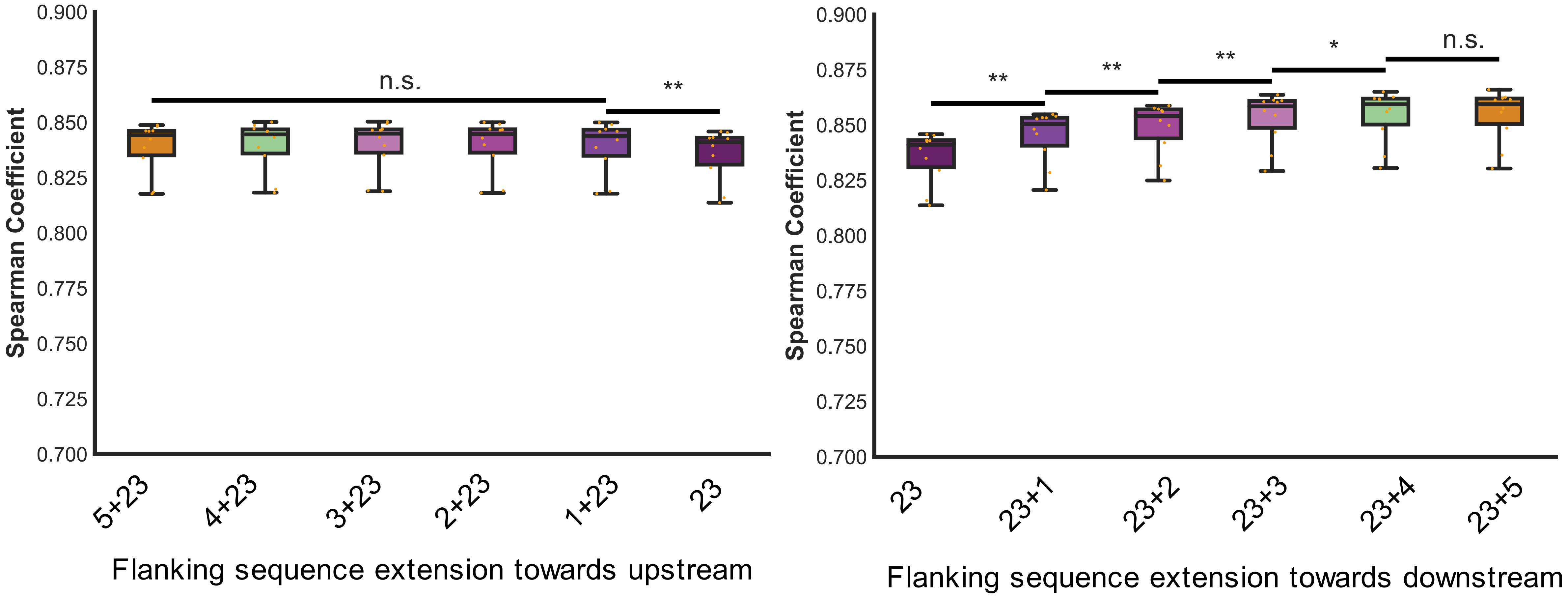

Supplementary Fig. S4

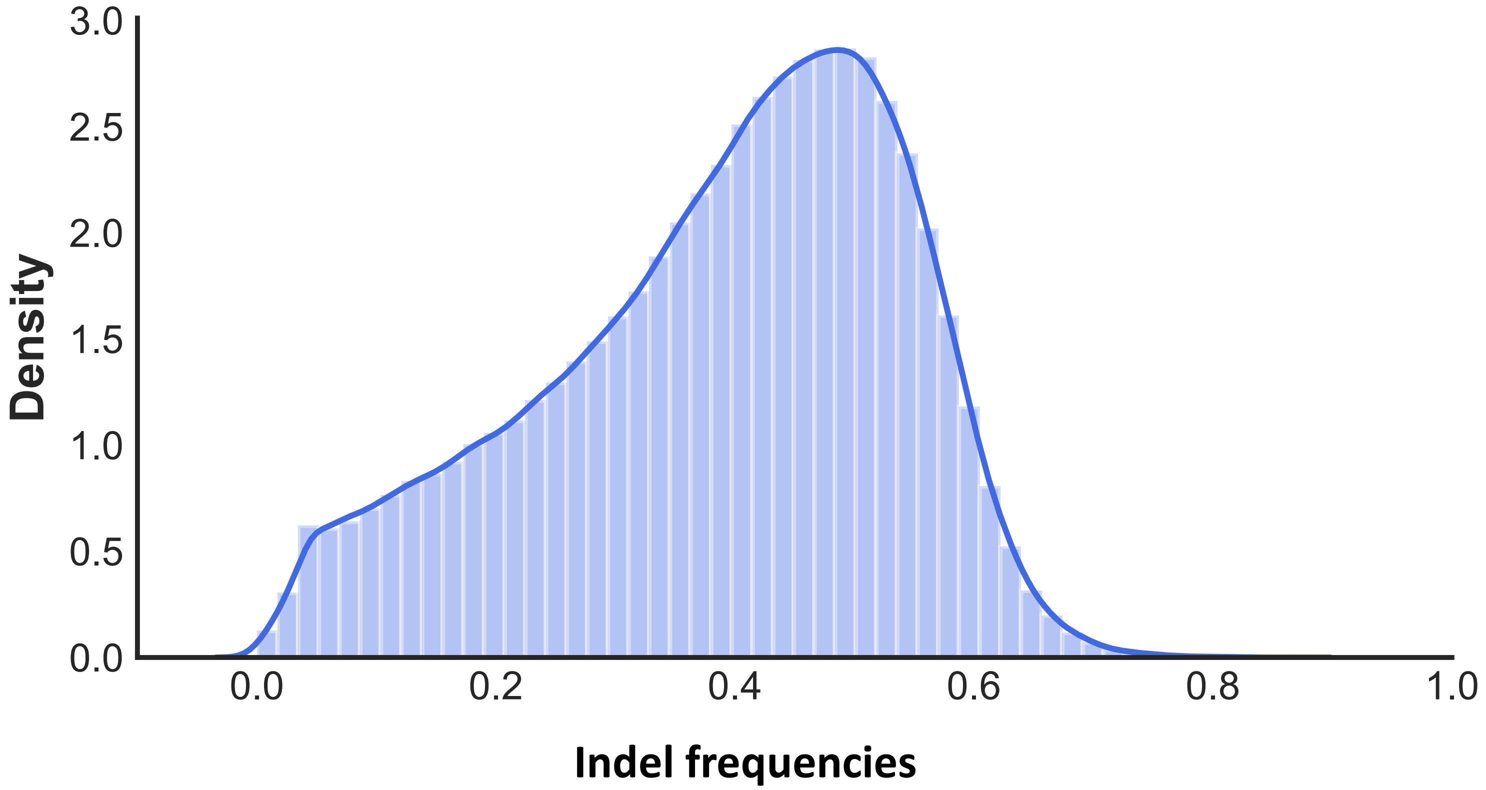

Supplementary Fig. S5

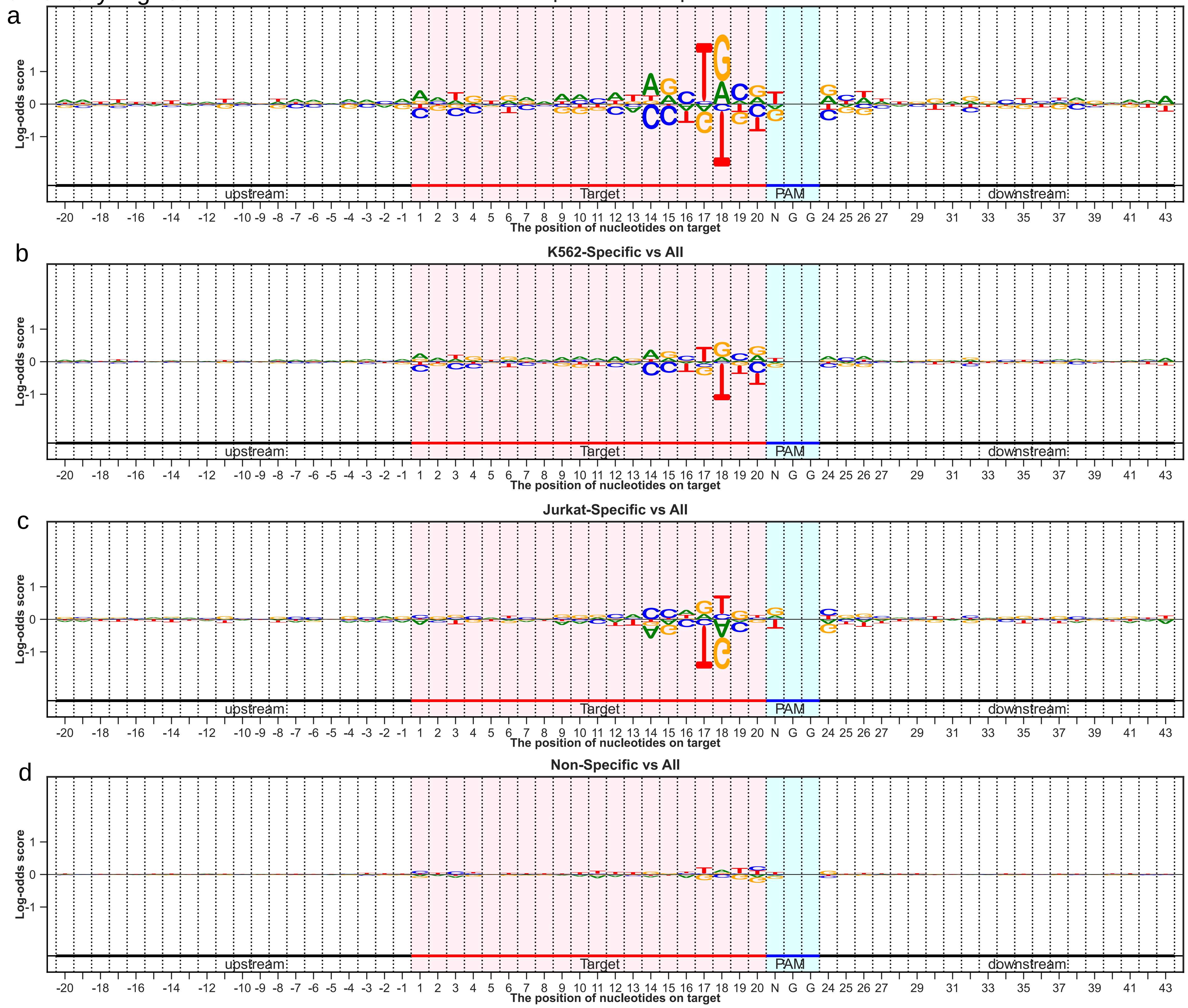

Supplementary Fig. S6

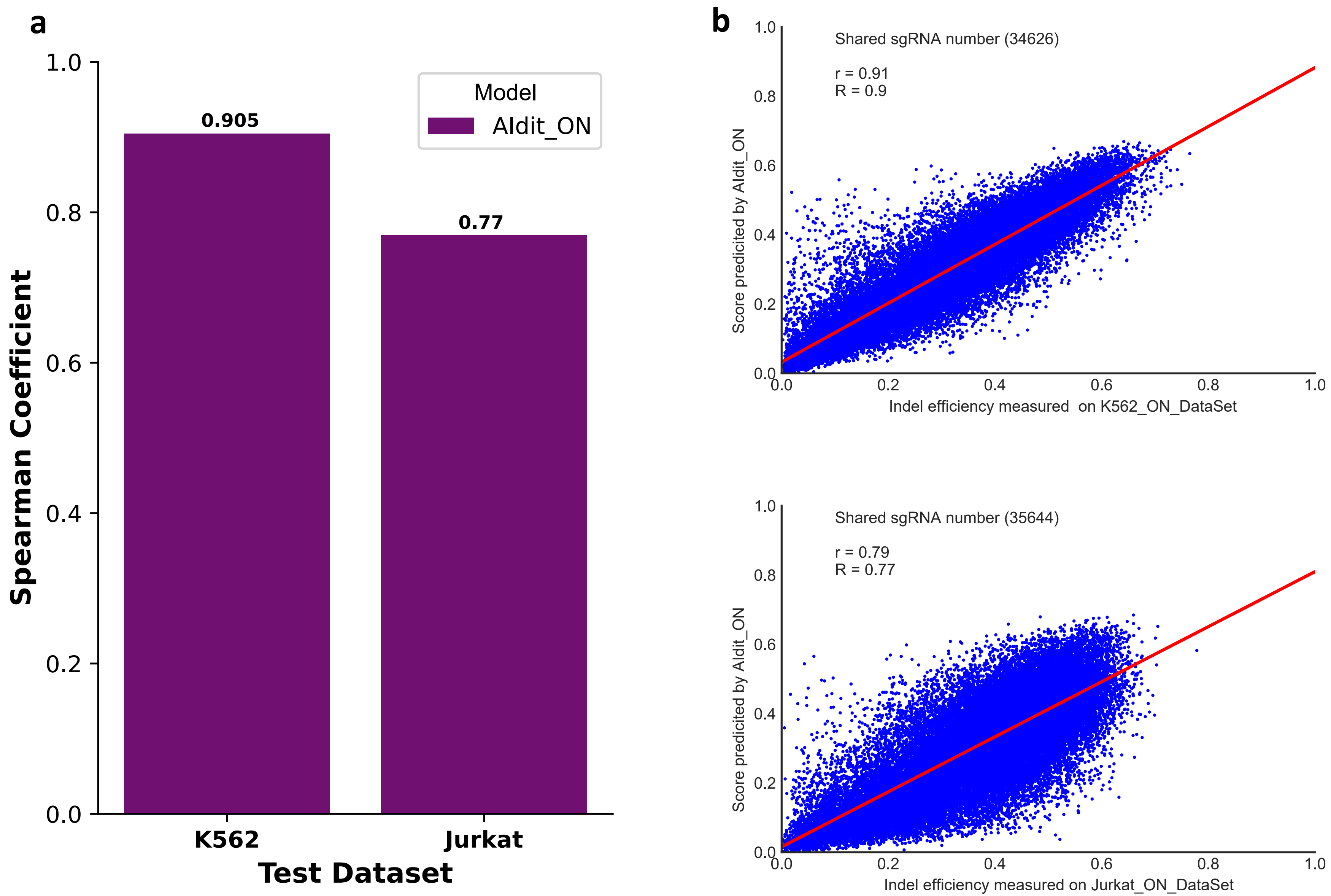

Supplementary Fig. S7

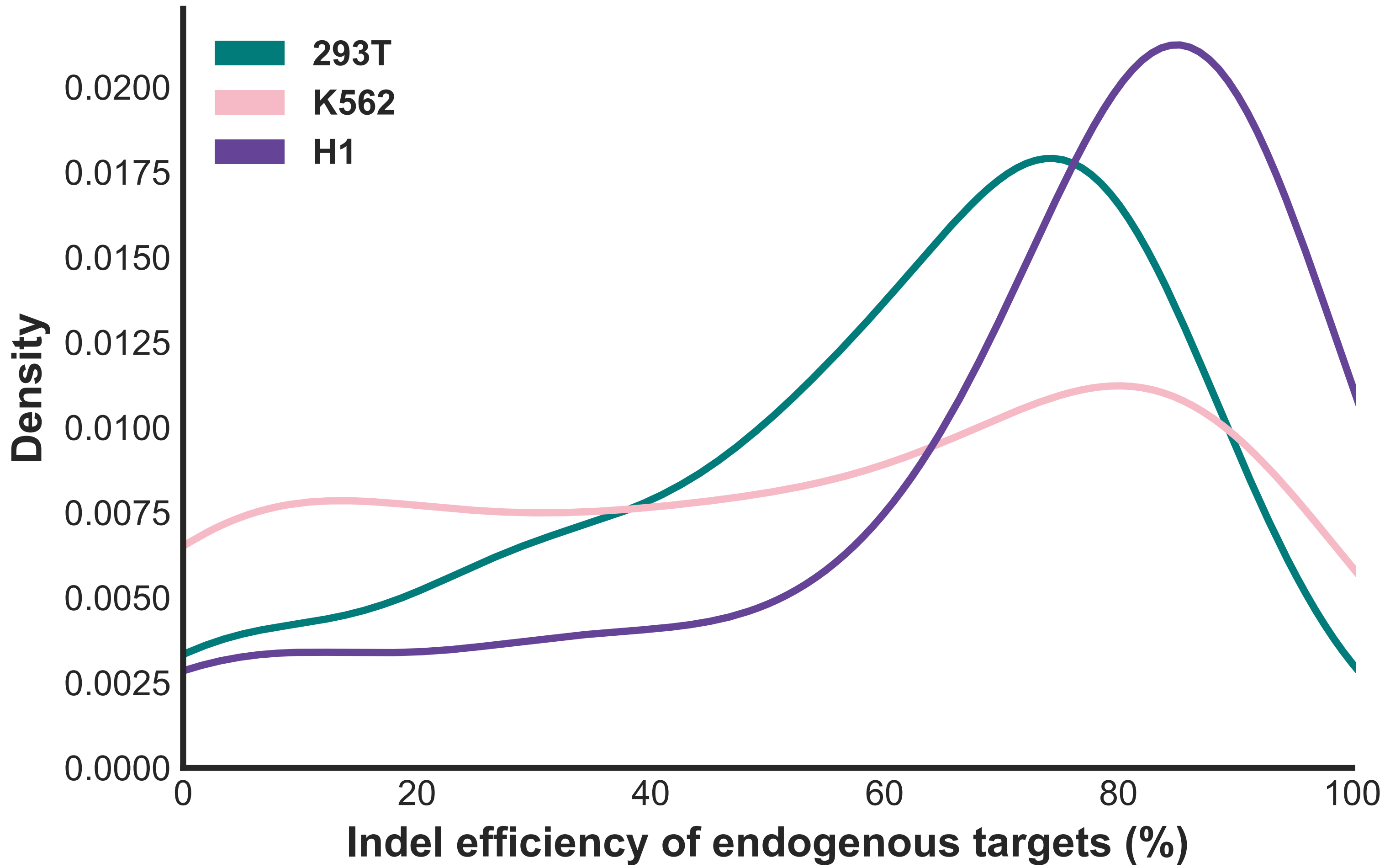

Supplementary Fig. S8

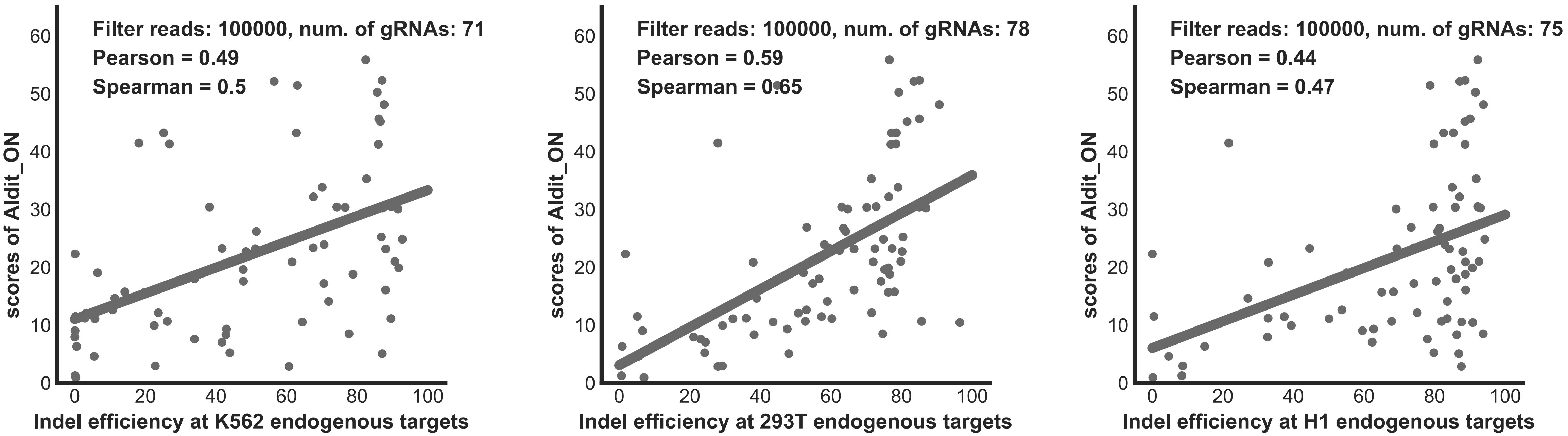

Supplementary Fig. S9

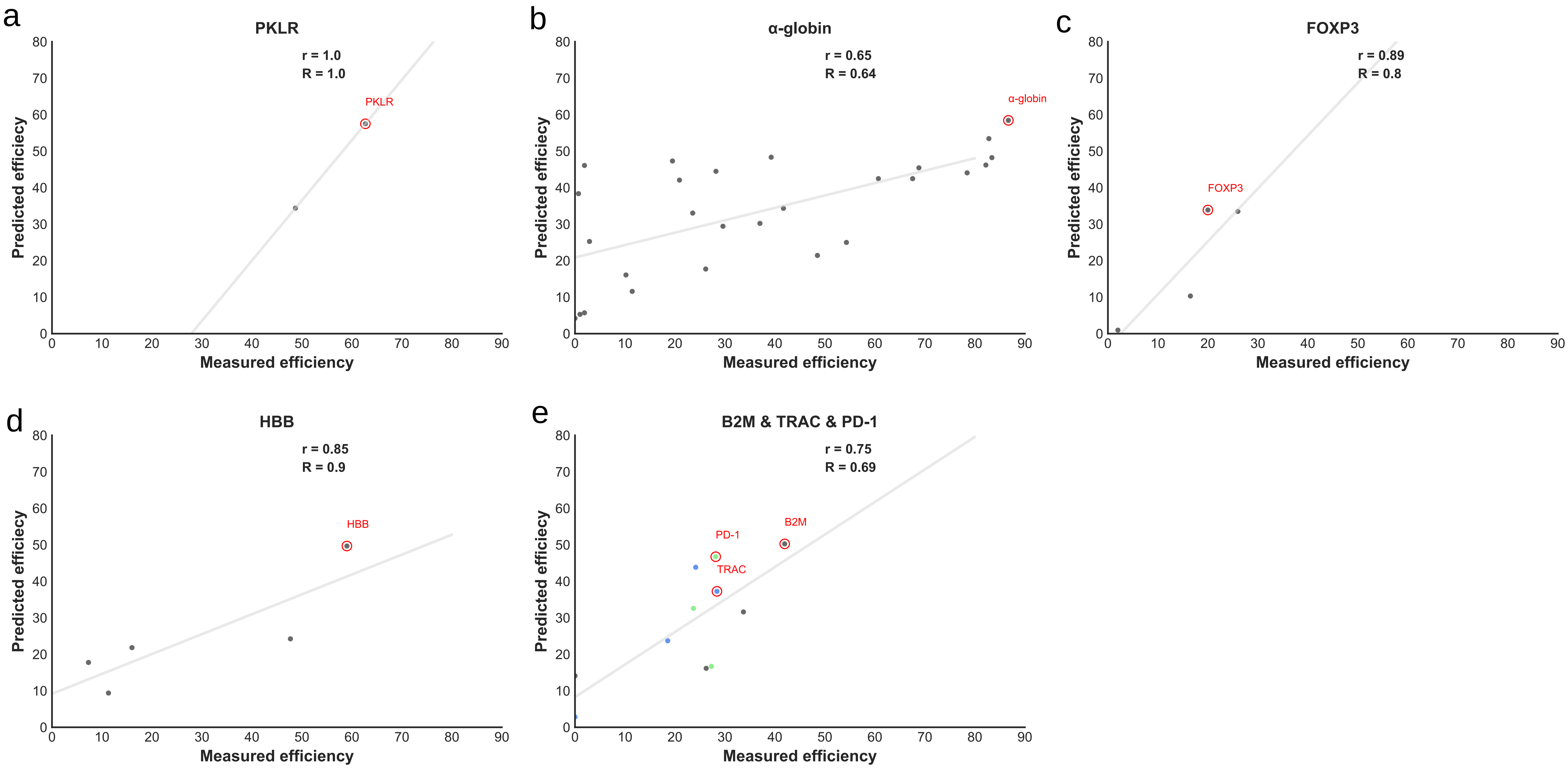

Supplementary Fig. S10

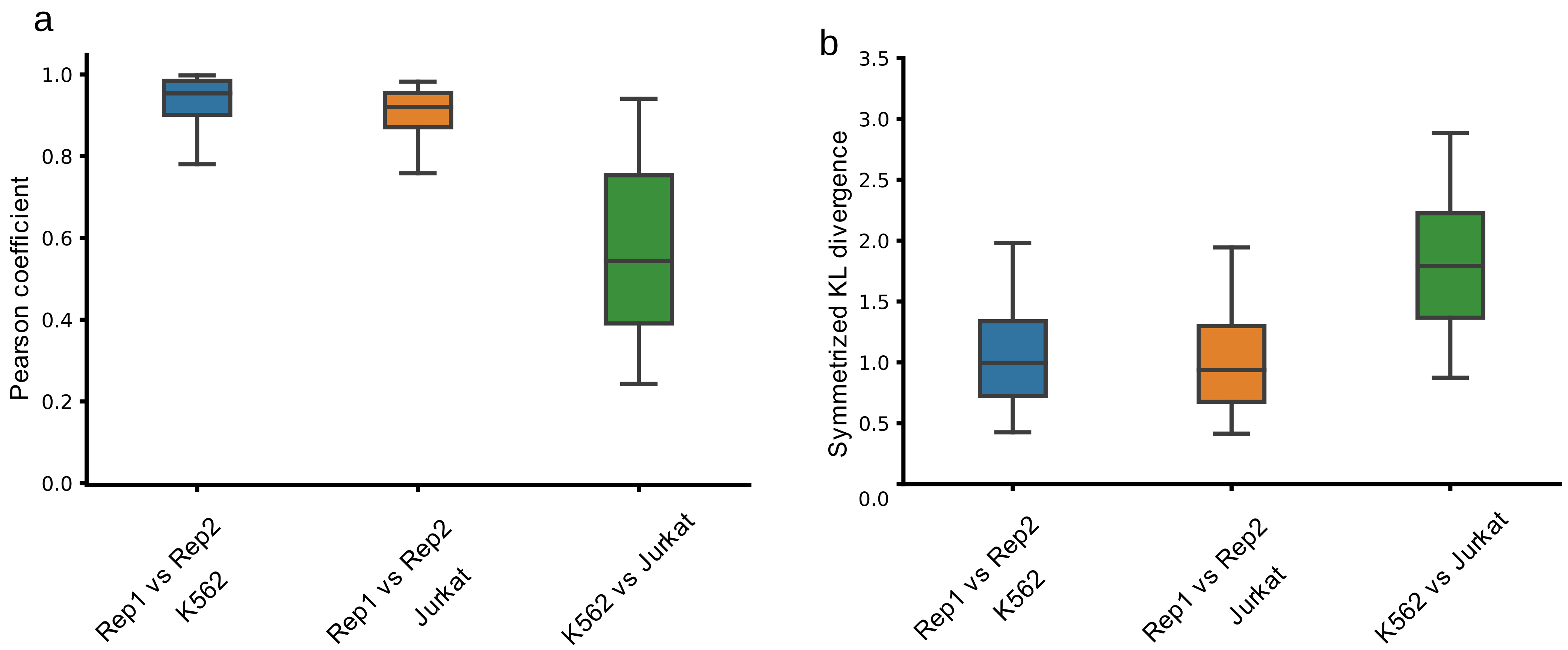

Supplementary Fig. S11

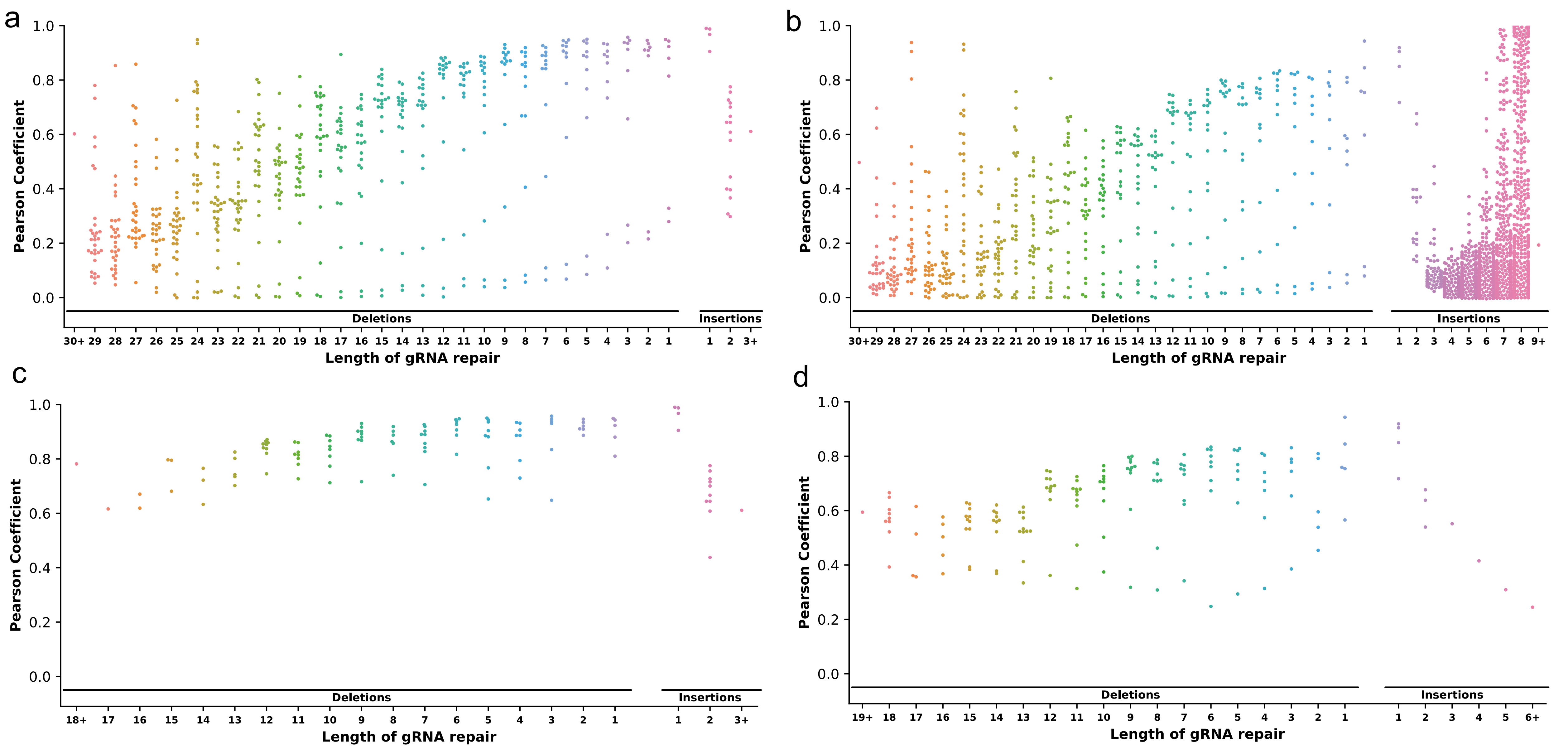

Supplementary Fig. S12

a

Predicted categories	Dataset	Model	Pearson	SymKL	MSE
K562(117)	K562	AIdit_DSB_K562	0.9443*	0.33164*	0.0001073*
		Lindel	0.86881	0.53044	0.0002695
		ForeCasT	0.71406	0.88041	0.0006574
	ForeCasT_Lindel	AIdit_DSB_K562	0.83626*	0.89639*	0.0003227*
		Lindel	0.82823	0.94591	0.0003312
		ForeCasT	0.71005	1.20965	0.0006523
	Jurkat	AIdit_DSB_K562	0.48059*	1.15008*	0.0009876
		Lindel	0.4367	1.27409	0.0009173*
		ForeCasT	0.23498	2.91095	0.0015991

*Best-in-class performance among different models in 3 datasets.

b

Predicted categories	Dataset	Model	Pearson	SymKL	MSE
Jurkat(163)	K562	AIdit_DSB_Jurkat	0.51733	1.20246	0.000712
		Lindel	0.87028*	0.55598*	0.000192*
		ForeCasT	0.71033	0.90641	0.000461
	ForeCasT_Lindel	AIdit_DSB_Jurkat	0.41359	1.88852	0.00087
		Lindel	0.82164*	0.98597*	0.000242*
		ForeCasT	0.69935	1.24992	0.000465
	Jurkat	AIdit_DSB_Jurkat	0.97230*	0.23704*	0.000055*
		Lindel	0.46677	1.30748	0.000638
		ForeCasT	0.24616	2.94097	0.00111

*Best-in-class performance among different models in 3 datasets.

Supplementary Fig. S13

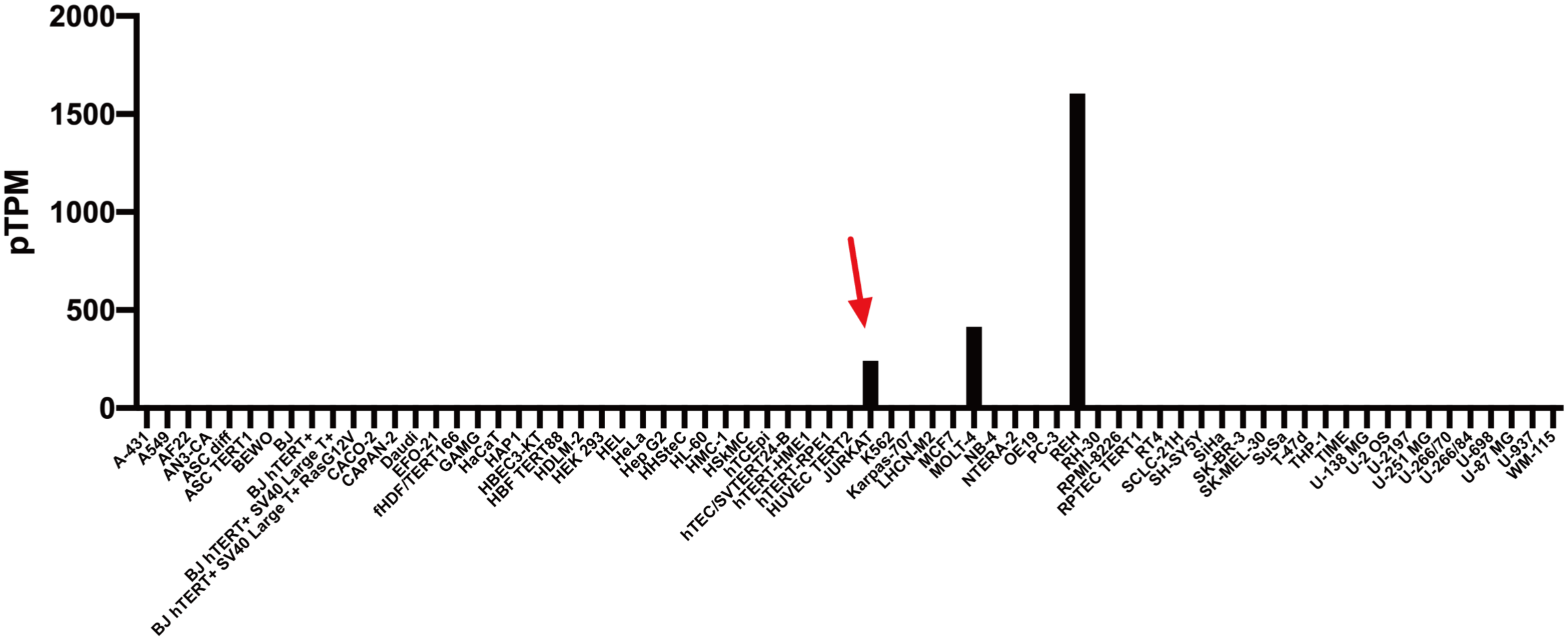

Supplementary Fig. S14

Mismatch number=1…7

a

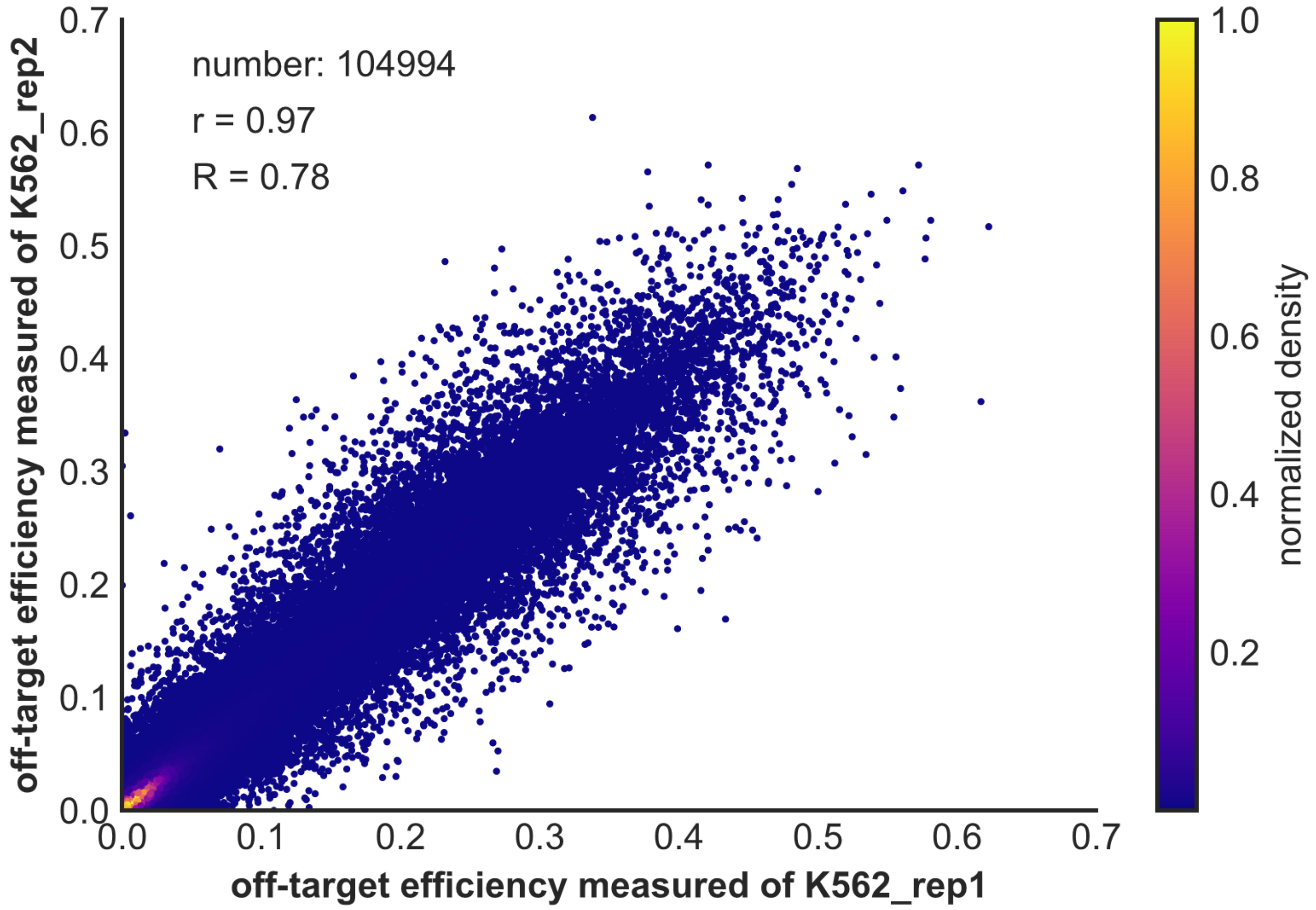

Supplementary Fig. S14

b

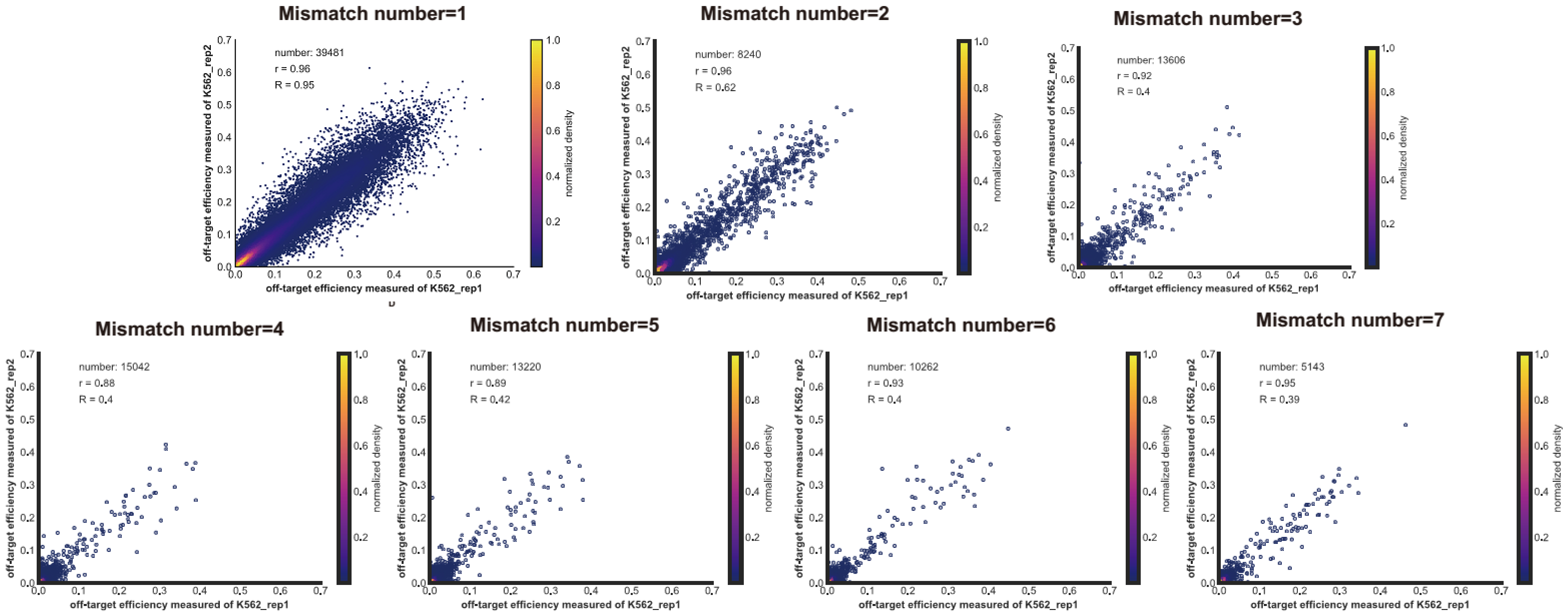

Supplementary Fig. S15

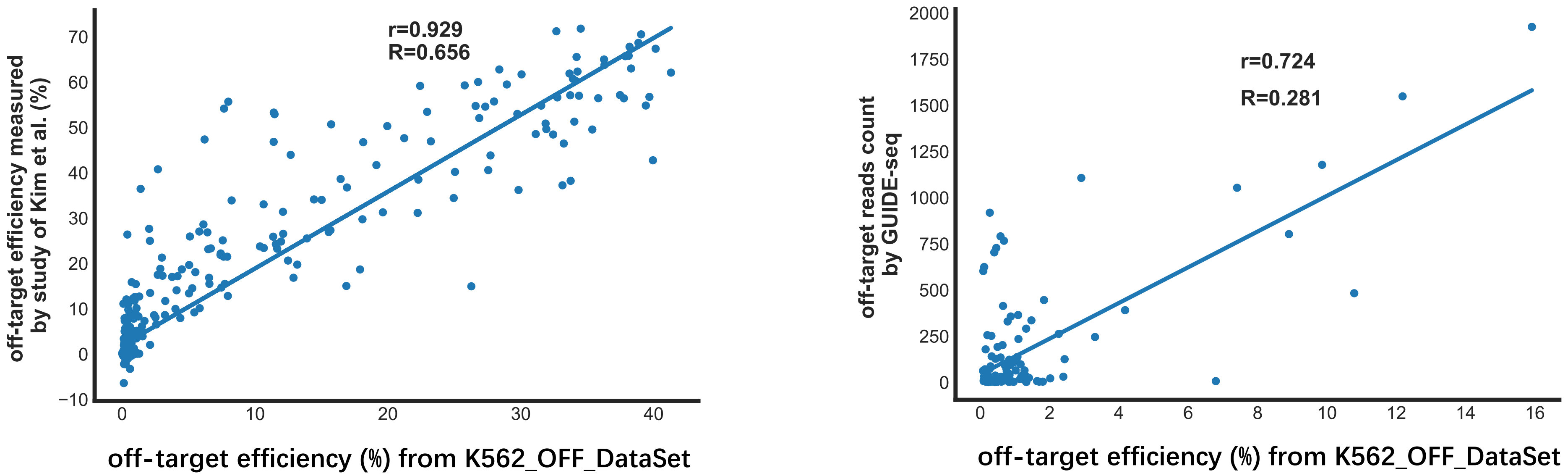

Supplementary Fig. S16

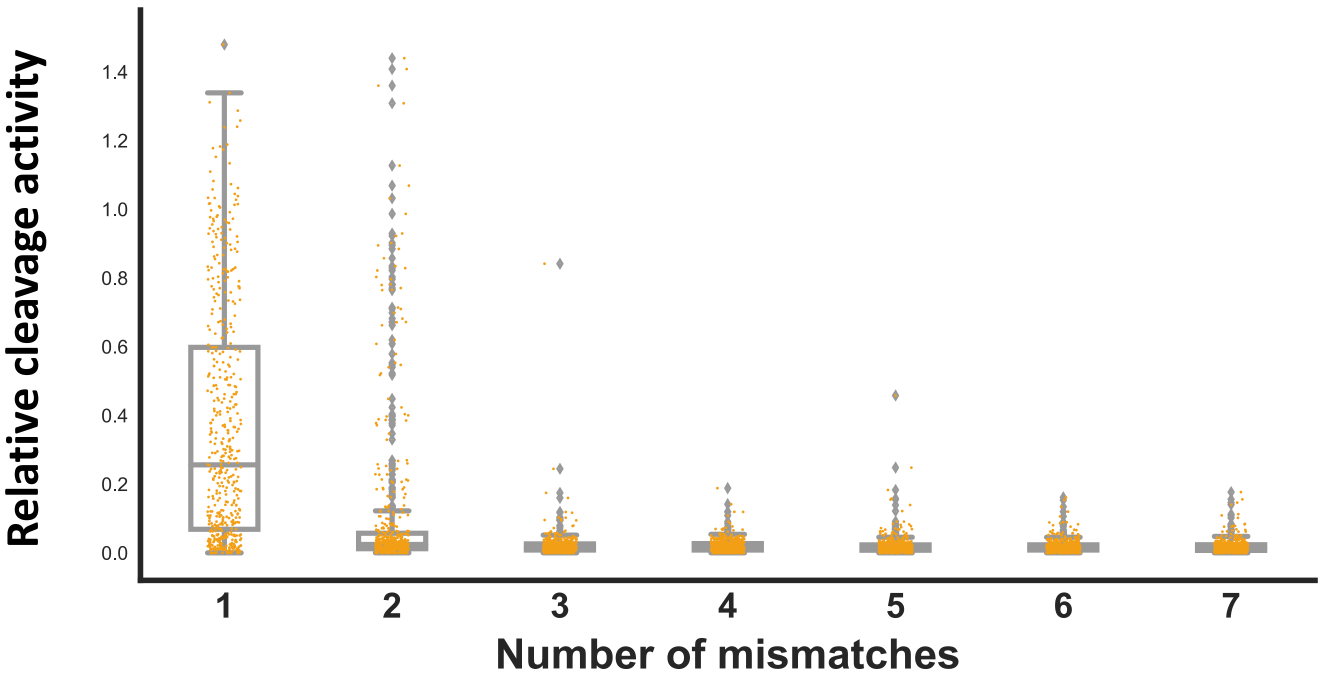

Supplementary Fig. S17

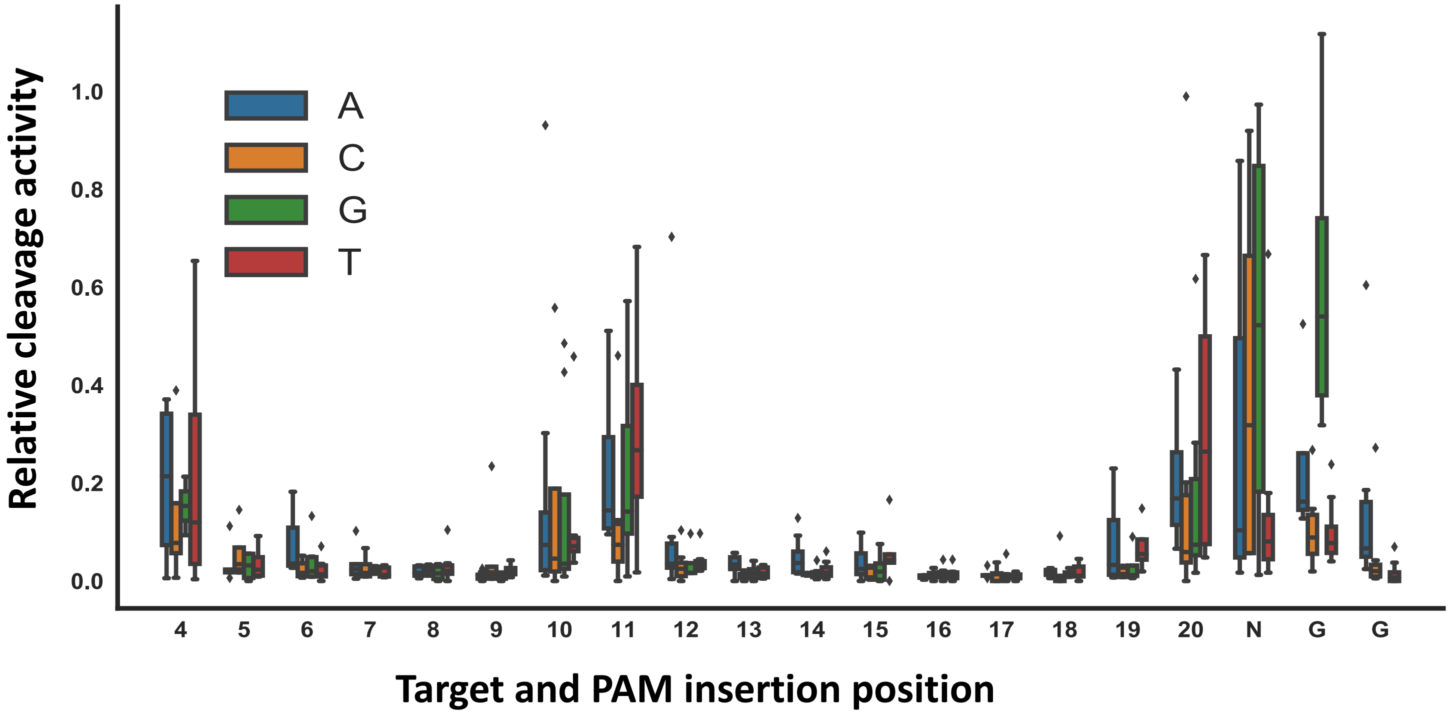

Supplementary Fig. S18

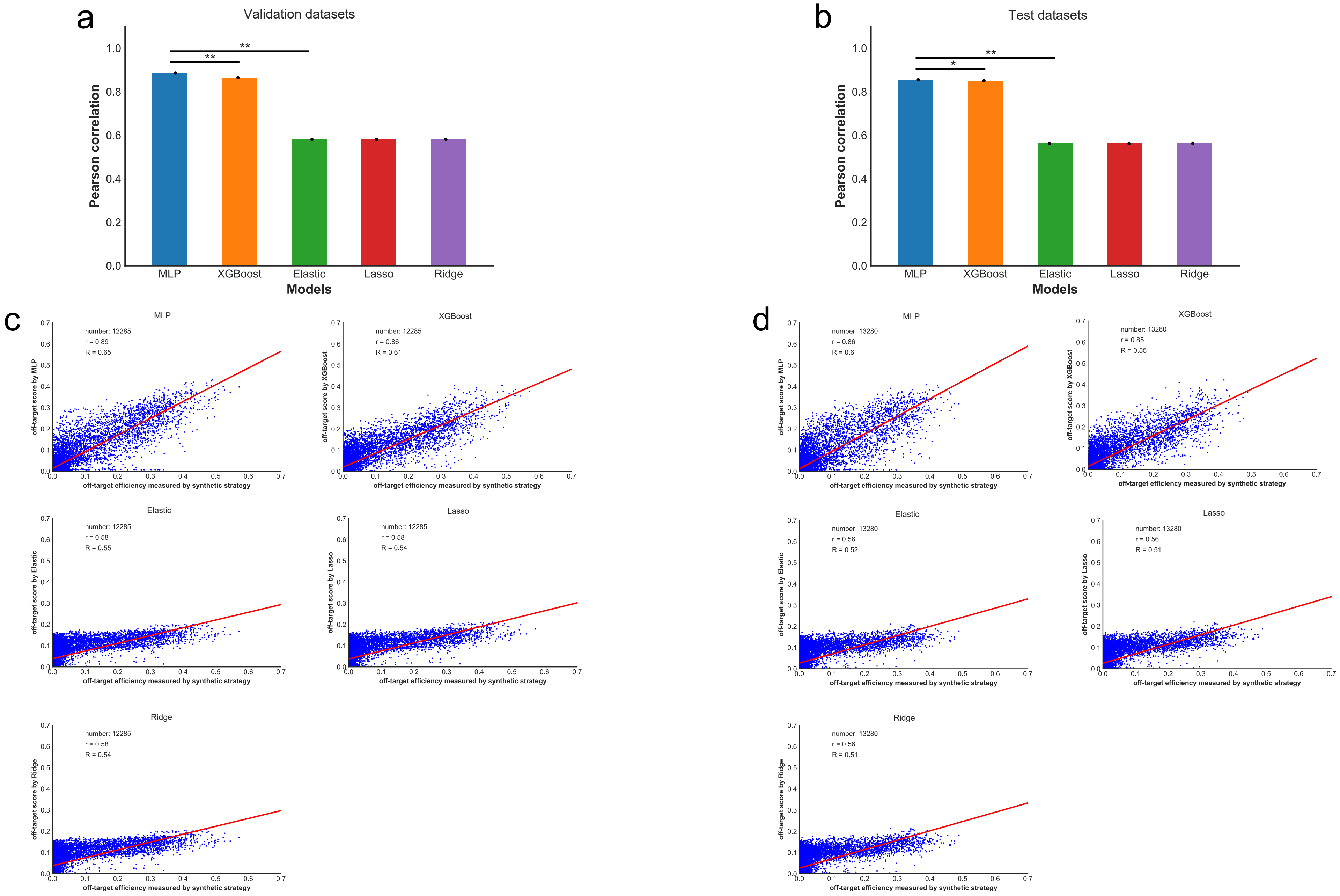

Supplementary Fig. S19

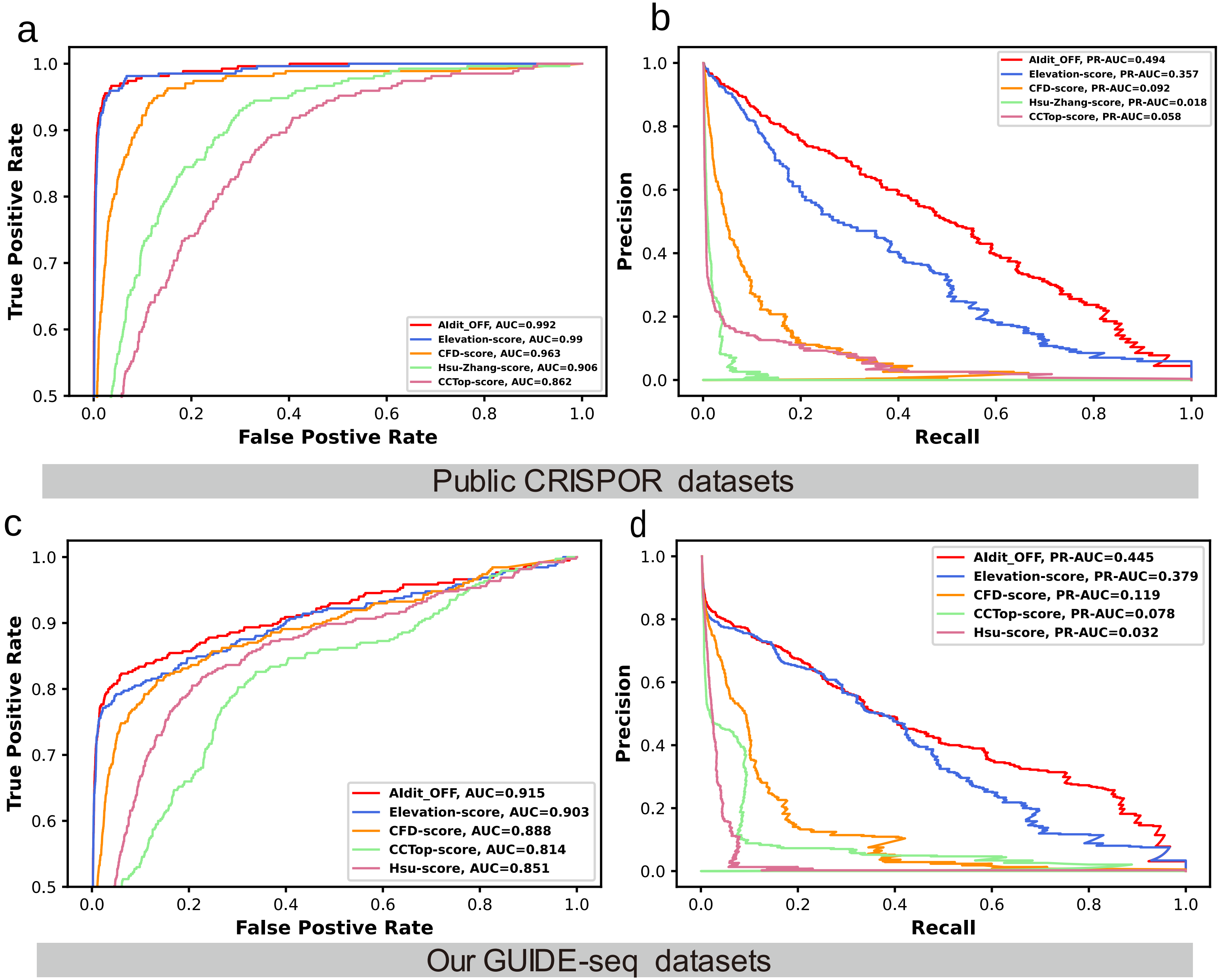

Supplementary Fig. S20

Dataset	No. of gRNA	No. of possible off-targets	No. of identified off-targets	ROC-AUC		PR-AUC		TPR		FPR		Precision	
				AIdit_OFF	CFD score	AIdit_OFF	CFD score	AIdit_OFF	CFD score	AIdit_OFF	CFD score	AIdit_OFF	CFD score
**GUIDE-seq (public)**	31	635527	262	0.996	0.974	0.468	0.083	99.237%	99.237%	10.279%	28.857%	0.397%	0.142%
**CRISPOR**	12	387973	270	0.992	0.963	0.494	0.092	98.148%	98.148%	11.236%	29.460%	0.605%	0.231%
**Tsai**	7	220140	198	0.994	0.978	0.561	0.187	98.990%	99.495%	15.243%	30.745%	0.581%	0.290%
**Kleinstiver**	5	140321	63	0.998	0.958	0.558	0.130	100.000%	98.413%	11.667%	30.142%	0.384%	0.146%
**Listgarten**	20	333185	29	0.999	0.983	0.281	0.045	100.000%	100.000%	6.694%	28.377%	0.130%	0.031%
**GUIDE-seq (this study)**	5	172901	385	0.915	0.888	0.445	0.119	88.052%	86.753%	27.362%	31.569%	0.713%	0.610%

Supplementary Fig. S21

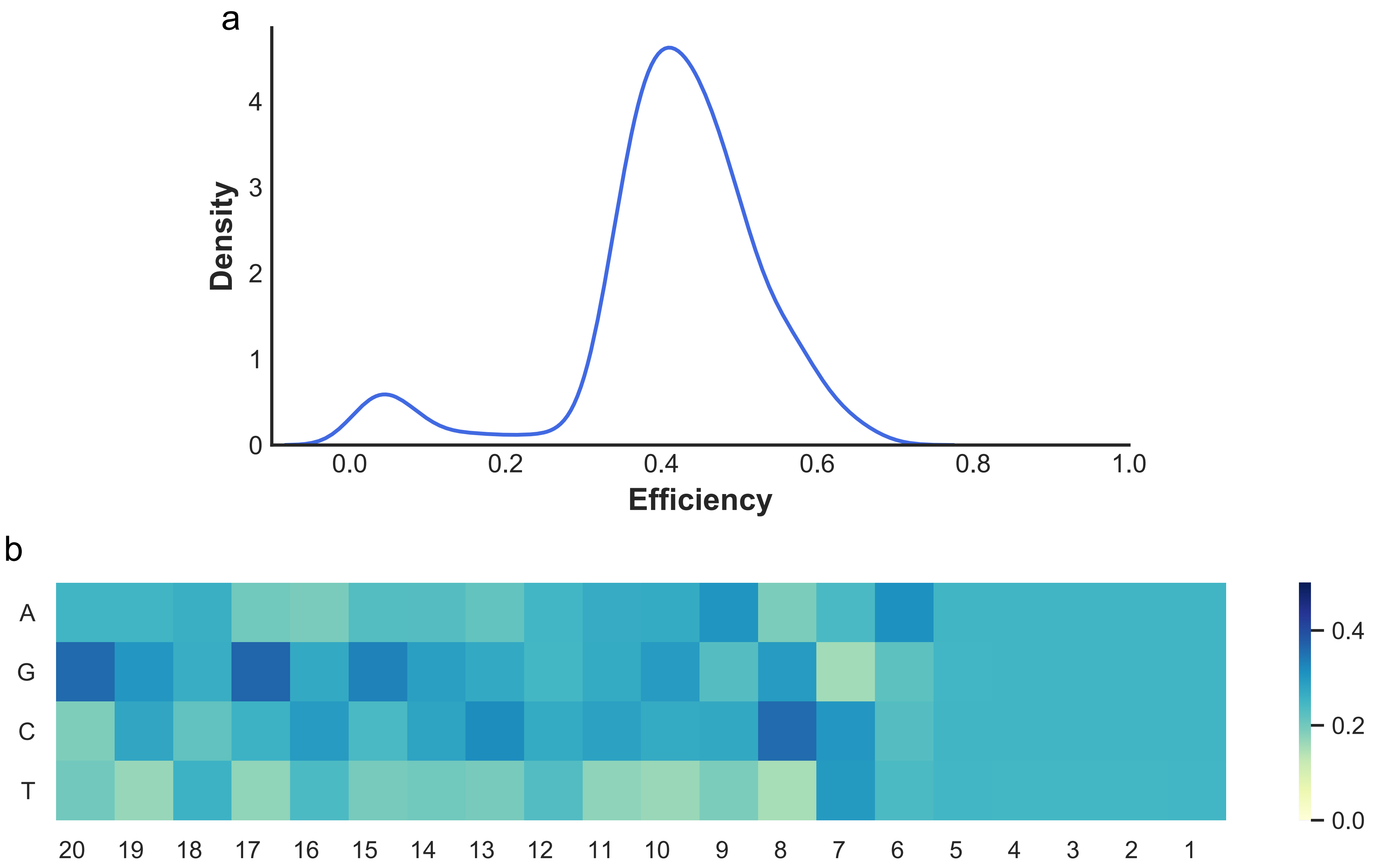

Supplementary Fig. S22

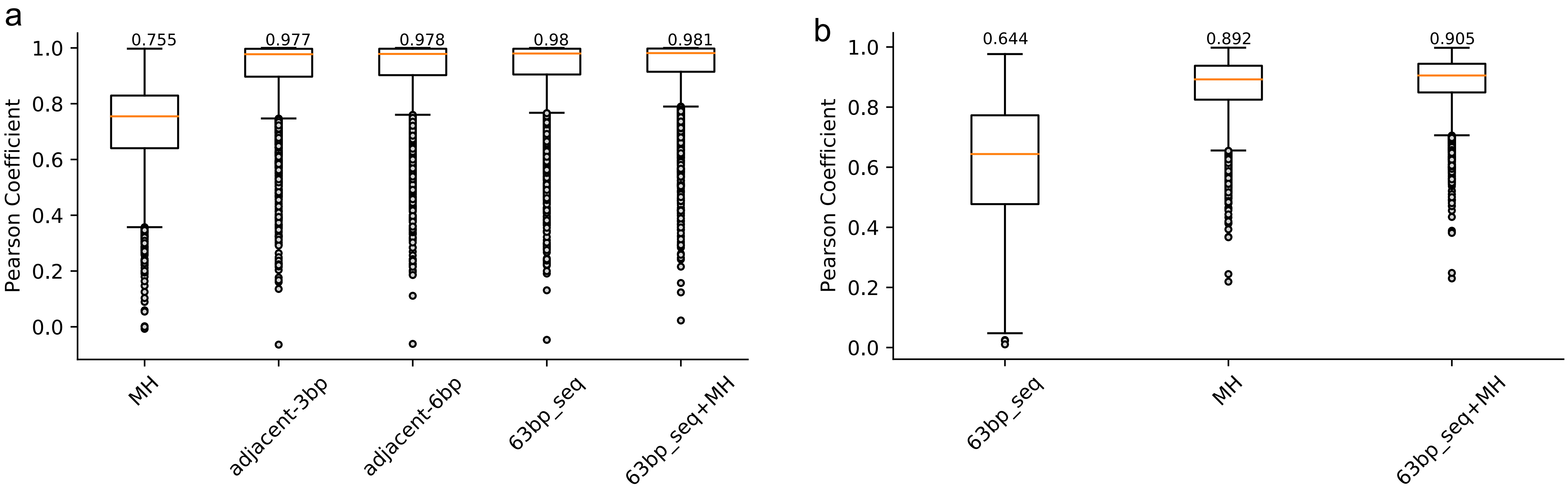

Supplement: Supplementary file 1 — Supplementary Fig merged [file 41421_2023_549_MOESM1_ESM.pdf]
