# Supplementary material for: Deep sampling of gRNA in the human genome and deep-learning-informed prediction of gRNA activities
Source: Cell Discov. 2023 May 16;9:48. doi: 10.1038/s41421-023-00549-9 (PMC10188485; doi:10.1038/s41421-023-00549-9)
Supplement: Supplementary file 2 — Supplementary Figure Legend [file 41421_2023_549_MOESM2_ESM.docx]

**Supplementary figure legends**

Supplementary Fig. S1 a. The distribution of indel frequencies in K562 by sub-pools. x-axis: indel frequencies. The gRNAs of the 740k library were synthesized from eight synthetic oligo pools, which were denoted as library-1 to library-8. Each of the synthetic oligo pool fits a maximal synthesis capacity of a GenScript 92K chip, and we synthesized eight oligo pools to achieve the 743,344 gRNAs in total. b. A heatmap showed the correlation between biological replicates of the SpCas9/gRNA cleavage activities quantified in K562 and Jurkat. The pearson correlations were color coded.

Supplementary Fig. S2. Comparison of prediction performances on cleavage activities of SpCas9/gRNA from different algorithms. The models were trained by 10-fold cross-validation and evaluated on testing dataset. Each dot represented the Spearman correlation between the measured indel frequencies and the predicted efficiencies from 10-fold cross-validation. *P<0.05 and **P<1e-5 show statistic significances between two deep learning-based approaches, between deep learning-based approaches and the best algorithm in conventional machine learning-based approaches using Steiger’s test.

Supplementary Fig. S3. The influence of input sequence length to model performance. The 10-fold cross-validation of the RNN models were performed on training datasets with 23 bp target sequences only (23) and with additional 1~5 bp upstream sequences (left panel). The 10-fold cross-validation of the RNN models were also performed on training datasets with 23 bp target sequences only (23) and with additional 1~5 bp downstream sequences (right panel). Each dot represented the Spearman correlation coefficients between the measured indel frequencies and the predicted efficiencies from the 10-fold cross-validation (*P<0.05 and **P<1e-5, Steiger’s test).

Supplementary Fig. S4. The distribution of indel frequencies in Jurkat. x-axis: indel frequencies.

Supplementary Fig. S5. Nucleotide preferences of gRNAs of the K562-Specific, Jurkat-Specific, and Non-Specific subgroups

Supplementary Fig. S6. Performance of cross prediction between training datasets and test datasets from AIdit_ON. Box plot(a) and dot plot(b) on independent test datasets from K562 and Jurkat. The Spearman correlation between measured and predicted indel frequencies was used as an evaluation metric.

Supplementary Fig. S7. The distribution of indel frequencies measured at endogenous sites individually in 293T, K562, and H1.

Supplementary Fig. S8. The generalization performance of AIdit_ON across three different cell lines. Both the Spearman and Pearson correlation coefficients are shown between AIdit_ON scores and the measured indel frequencies at endogenous sites for K562 (left), 293T (middle) and H1 (right), respectively.

Supplementary Fig. S9. The AIdit_ON model predicts SpCas9/gRNA cleavage activities in human primary cells for CRISPR therapy. Each red circle represents the gRNA that they ultimately chose for further experiments.

Supplementary Fig. S10. Box plots showed the reproducibility of DSB-induced repair outcomes between two biological replicates of each of the two cell lines (K562 and Jurkat) and between the two cell lines. Pearson coefficient (a) and Symmetrized KL divergence (b) were used as metrics.

Supplementary Fig. S11. Scattered plots showed the reproducibility of DSB-induced repair outcomes between two biological replicates of each of the two cell lines before and after aggregating the repairing categories. (a) 631 original categories in K562; (b) 610 original deletion categories, and all possible 1-8 bp insertion categories and one insertion more than 9 bp in Jurkat; (c) 117 aggregated categories in K562; (d) 163 aggregated categories in Jurkat.

Supplementary Fig. S12. The evaluation of generalization performance on prediction of repair outcomes. (a) AIdit_DSB_K562, Lindel, and ForeCasT models on the K562, ForeCast_Lindel, and Jurkat datasets using the 117 aggregated repair categories; (b) AIdit_DSB_Jurkat, Lindel, and ForeCasT models on the K562, ForeCast_Lindel, and Jurkat datasets using the 163 aggregated repair categories. Asteria indicated the best performed models in each benchmarking based on various metrics.

Supplementary Fig. S13. The RNA expression level of DNTT across different cell lines. This RNA-seq data is obtained from The Human Protein Atlas (HPA) database at https://www.proteinatlas.org/.

Supplementary Fig. S14. Scattered plots showed the reproducibility of the cleavage activities of spCas9/gRNA on all off-target sequences (a) or off-target sequences with different numbers of mismatches (b) between two biological replicates in K562.

Supplementary Fig. S15. Scattered plots showed the pearson correlation(r) and spearman correlation(R) of indel frequencies between quantifications in our study and in Kim et al (a), and between quantifications in our study and from GUIDE-seq (b).

Supplementary Fig. S16. The influence of the number of mismatches on off-target sequences. The relative cleavage activity, which is a relative ratio of indel efficiencies between the off-target sequences and the corresponding matched targets, were plotted in y-axis.

Supplementary Fig. S17. The influence of insertion position on off-target sequences with 1bp bulge. The relative cleavage activity, which is a relative ratio of indel efficiencies between the off-target sequences and the corresponding matched targets, were plotted for each nucleotide at each position in y-axis. Positions of 1-3 are excluded from this analysis due to data filtering.

Supplementary Fig. S18. The evaluation of model performances on prediction of cleavage activities on synthetic off-target sites. (a) box plot on validation datasets; (b)box plot on testing datasets. (c) dot plot on validation datasets; (d) dot plot on testing datasets.

Supplementary Fig. S19. Comparison of model performances on predicting cleavage activities on off-target sequences. The benchmark was conducted on endogenous off-target datasets from public CRISPOR datasets (a-b) and our GUIDE-seq datasets (c-d) across different models (AIdit_OFF, Elevation_score, CFD-score, CCTop-score, and Hsu-score). Two metrics were compared, including AUC (a, c) to examine the false positive rate and PR-AUC (b, d) to examine the recall rate.

Supplementary Fig. S20. Evaluation of model generalization performance between the AIdit_OFF model and the CFD model, based on the minimal off-target scores from each model, which is 0.0069 for the AIdit_OFF model and 0.023 for the CFD model.

Supplementary Fig. S21. (a) Distribution of on-target indel frequencies of the Type III group of the 180k library. (b) Heatmap of nucleotide distribution of gRNA of the Type III group.

Supplementary Fig. S22. Effect of the target sequence and microhomology features on prediction of SpCas9-induced DSB repair outcomes. (a) Feature evaluation on prediction models for the insertion repair outcomes. The x-axis showed the training feature sets of the microhomology, 3-bp, and 6-bp sequence around the cleavage site, the 63-bp sequence and the combined features of 63-bp sequence and microhomology, respectively. The Pearson correlation coefficients between insertion outcomes measured by experiments and scores predicted by models at each target sequence are shown. (b) Feature evaluation of predictions models on the deletion repair outcomes. Feature sets used to train these models include the 63-bp sequence, the microhomology, the combination of the sequence and microhomology, respectively. The y-axis shows the Pearson correlation coefficients between the deletion outcomes measured and scores predicted by models at each target sequence.
